# Therapies for Chronic Spontaneous Urticaria: Present and Future Developments

**DOI:** 10.3390/ph17111499

**Published:** 2024-11-07

**Authors:** Riccardo Asero, Paolo Calzari, Silvia Vaienti, Massimo Cugno

**Affiliations:** 1 Clinica San Carlo, Ambulatorio di Allergologia, 20037 Paderno Dugnano, Italy; r.asero@libero.it; 2 Department of Pathophysiology and Transplantation, Scuola di Specializzazione, Allergologia e Immunologia Clinica, Università degli Studi di Milano, 20122 Milan, Italy; paolo.calzari@unimi.it; 3 Section of Dermatology and Venereology, Department of Medicine, University of Verona, 37126 Verona, Italy; silvia.vaienti@hotmail.it; 4 Department of Pathophysiology and Transplantation, Internal Medicine, Fondazione IRCCS Ca’ Granda Ospedale Maggiore Policlinico, Università degli Studi di Milano, 20122 Milan, Italy

**Keywords:** chronic spontaneous urticaria, antihistamines, omalizumab, corticosteroids, cyclosporin, dupilumab, barzolvolimab, CDX-0159, Tezepelumab, vixarelimab, mepolizumab, UB-221, remibrutinib, rilzabrutinib, TAS 5315, TLL-018, povorcitinib, EP 262

## Abstract

Chronic spontaneous urticaria (CSU) is a complex dermatological condition characterized by recurrent wheals and/or angioedema lasting for more than six weeks, significantly impairing patients’ quality of life. According to European guidelines, the first step in treatment involves second-generation H1-antihistamines (sgAHs), which block peripheral H1 receptors to alleviate symptoms. In cases with inadequate responses, the dose of antihistamines can be increased by up to fourfold. If symptoms persist despite this adjustment, the next step involves the use of omalizumab, a monoclonal anti-IgE antibody, which has shown efficacy in the majority of cases. However, a subset of patients remains refractory, necessitating alternative treatments such as immunosuppressive agents like cyclosporine or azathioprine. To address these unmet needs, several new therapeutic targets are being explored. Among them, significant attention is being given to drugs that block Bruton’s tyrosine kinase (BTK), such as remibrutinib, which reduces mast cell activation. Therapies like dupilumab, which target the interleukin-4 (IL-4) and IL-13 pathways, are also under investigation. Additionally, molecules targeting the Mas-related G protein-coupled receptor X2 (MRGPRX2), and those inhibiting the tyrosine kinase receptor Kit, such as barzolvolimab, show promise in clinical studies. These emerging treatments offer new options for patients with difficult-to-treat CSU and have the potential to modify the natural course of the disease by targeting key immune pathways, helping to achieve longer-term remission. Further research is essential to better elucidate the pathophysiology of CSU and optimize treatment protocols to achieve long-term benefits in managing this condition. Altogether, the future of CSU treatments that target pathogenetic mechanisms seems promising.

## 1. Introduction

Urticaria is clinically characterized by the appearance of wheals (hives), which can be accompanied by angioedema. When the spontaneous recurrence of short-lived wheals, angioedema, or both lasts for more than 6 weeks, this is defined as chronic spontaneous urticaria (CSU) [1]. CSU has an estimated prevalence of 0.5% to 1% and female predominance (female/male ratio: 2/1) [2]. CSU symptoms significantly affect many aspects of patients’ health-related quality of life, and the health status scores of CSU patients have been reported to be comparable with those of patients with coronary artery disease in terms of work performance, sleep disruption, emotional reactions, and social interactions [3]. Evaluating CSU can be difficult, and the Weekly Urticaria Activity Score (UAS7) is one of the most commonly used methods to accomplish this. This tool relies on the patient’s daily self-assessment of key symptoms, including wheals and itching. Each symptom is rated on a scale from 0 to 3, where 0 indicates no intensity and 3 represents severe intensity. Patients record their scores every day for seven days, with the total score ranging from 0 to 42 [1]. The international guidelines recommend treating patients until a complete clinical response is achieved. Currently, a three-step approach is suggested. The first step consists of using second-generation H1-antihistamines (sgAHs) at the licensed dosage. The second step involves increasing the use of sgAHs up to four times the licensed dosage. The third step consists of the addition of the anti-IgE monoclonal antibody, omalizumab [1] (Table 1). Although most patients with CSU achieve complete or partial control of the disease with stepwise treatment, some remain unresponsive. In such cases, switching to immunosuppressive therapy is suggested, with cyclosporine being the most studied and frequently prescribed option. Azathioprine, methotrexate (MTX) and mycophenolate mofetil (MMF) have also been proposed as alternative treatments. In the case of acute exacerbation, the guidelines suggest considering a short course of systemic glucocorticosteroids. Other therapies are being developed to target the pathomechanisms of CSU. In particular, these target the mast cells [4], whose activation leads to wheal formation via the release of vasoactive substances such as histamine. The sequence of events responsible for mast cell activation is not completely defined and involves autoimmunity [5,6], autoallergy [7,8,9], the complement system, and coagulation [10,11,12,13], with the participation of other cells [14] such as eosinophils, endothelial cells, basophils, B lymphocytes, T lymphocytes, and monocytes.

An improvement in the understanding of CSU pathogenesis and the consequent development of new therapies has led to the definition of disease-modifying treatments (DMTs). These treatments are designed not only to alleviate symptoms but also to alter the underlying mechanisms driving the disease. DMTs aim to prevent or delay disease progression, achieve long-lasting remission without ongoing therapy, and directly target the core disease mechanisms.

DMTs include therapies that reduce the production of autoantibodies or target specific cytokines involved in inflammation and symptom manifestation: these drugs are reported in Table 2 (monoclonal antibodies) and in Table 3 (small molecules) [15].

**Figure 1:**
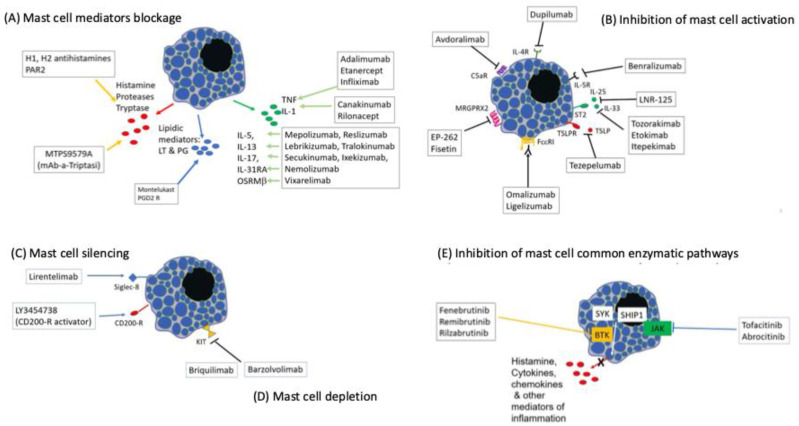
Mechanisms of action of currently used and potentially effective drugs in chronic spontaneous urticaria treatment.BTK: Bruton tyrosine kinase; C5aR: C5 a receptor; CD200 R: CD 200 receptor; FcεRI: high-affinity receptor for the fragment crystallizable region of immunoglobulin E; IL: Interleukin; IL 4R: interleukin 4 receptor; IL 5R: interleukin 5 receptor; IL 31RA: interleukin 31 receptor A; JAK: Janus kinase; Kit: tyrosine kinase receptor Kit; LT: leukotriene; mAb: monoclonal antibody; MRGPRX2: Mas-related G protein–coupled receptor X2; OSMRβ: Oncostatin-M specific receptor subunit beta; PAR2: Protease activated receptor 2; PG: prostaglandins, PGD2R: Prostaglandin D receptor; Siglec-8: Sialic acid-binding Ig-like lectin 8; SHIP 1: Src homology 2 (SH2) domain containing inositol polyphosphate 5-phosphatase 1; SYK: Tyrosine-protein kinase SYK; ST2: suppression of tumorigenicity 2; TNF: tumor necrosis factor; TSLP: thymic stromal lymphopoietin.

Here, we review the current and potential treatments for CSU based on the latest understanding of its pathogenesis. The therapeutic approaches are categorized according to the mechanisms of action of the different drugs: blocking mast cell mediators, inhibiting mast cell activation, silencing mast cells, depleting mast cells, targeting shared enzymatic pathways (Figure 1), and possibly using miscellaneous immunosuppressants.

### Search Methodology

The PubMed and Google Scholar databases were screened using ‘chronic spontaneous urticaria’ and ‘treatment’, examining research published between 1990 and 2024. We also searched the ClinicalTrials.gov database for recent and ongoing randomized clinical trials in CSU using the keyword “chronic spontaneous urticaria”.

BTK: Bruton tyrosine kinase; C5aR: C5 a receptor; CD200 R: CD 200 receptor; FcεRI: high-affinity receptor for the fragment crystallizable region of immunoglobulin E; IL: interleukin; IL 4R: interleukin 4 receptor; IL 5R: interleukin 5 receptor; IL 31RA: interleukin 31 receptor A; JAK: Janus kinase; Kit: tyrosine kinase receptor Kit; LT: leukotriene; mAb: monoclonal antibody; MRGPRX2: Mas-related G protein-coupled receptor X2; OSMRβ: oncostatin M-specific receptor subunit beta; PAR2: protease-activated receptor 2; PG: prostaglandins, PGD2R: prostaglandin D receptor; Siglec-8: sialic acid-binding Ig-like lectin 8; SHIP 1: Src homology 2 (SH2) domain containing inositol polyphosphate 5-phosphatase 1; SYK: tyrosine-protein kinase SYK; ST2: suppression of tumorigenicity 2; TNF: tumor necrosis factor; TSLP: thymic stromal lymphopoietin.

## 2. Mast Cell Mediators Blockage

### 2.1. Second-Generation H1-Antihistamines

The use of SgAHs is the first step in the treatment of CSU [1]. Their mechanism of action involves the highly selective blockade of peripheral H1 receptors (a G protein-coupled receptor—GPCR), preventing the histamine effectives of vasodilation and an increase in vascular permeability [27,28]. The blockade of peripheral H1 receptors occurs through inverse regulation, achieved by positioning a common phenyl group within the hydrophobic cavity [28]. Secondary ligand-binding sites in H1R, characterized by several polar residues, are novel targets which could be effectively blocked using optimized derivative groups [28].

The molecules most used to treat CSU are as follows: ebastine, bilastine, loratadine, desloratadine, rupatadine, cetirizine, and levocetirizine [1]. Compared to first-generation antihistamines, these drugs have a better therapeutic profile, selectively blocking peripheral histamine receptors, which leads to fewer side effects such as sedation and anticholinergic effects [29].

The choice between various antihistamines in CSU treatment often depends on the patient’s profile and preferences, as there is no conclusive clinical evidence demonstrating significant differences in terms of their efficacy [30,31,32]. However, a recent Indian study found that bilastine caused a more significant reduction in the mean total symptom score (MTSS) and pruritus scale within one week of administration compared to cetirizine. Moreover, bilastine had fewer sedative side effects than cetirizine, making it a preferable option for many patients [33]. Another study compared bilastine, fexofenadine, and levocetirizine in terms of treating CSU. At week 4, bilastine demonstrated a statistically significant improvement in urticaria symptoms compared to levocetirizine (*p* < 0.05). Additionally, bilastine enhanced the quality of life (QoL) of patients, as measured by the CU-Q2oL questionnaire (*p* < 0.05), significantly more than both fexofenadine and levocetirizine. Bilastine was also associated with significantly lower somnolence compared to fexofenadine and levocetirizine, even after up-dosing (*p* < 0.05). Regarding adverse events, bilastine had the fewest, with the most common being sedation, headache, nausea, and fatigue [34]. These findings are further supported by another study, showing a significant reduction in UAS7 scores in subjects treated with bilastine compared to those treated with levocetirizine (*p* = 0.03) [35].

In the case of standard doses of sgAHs lacking efficacy, guidelines allow the use of up to fourfold standard doses [1]. Studies have demonstrated the safety and efficacy of off-label high-dose sgAH therapy, including the use of bilastine, cetirizine, desloratadine, ebastine, fexofenadine, levocetirizine, and rupatadine, at doses up to four times the recommended daily amount [36,37,38,39,40,41]. However, increased doses of sgAHs are associated with a higher risk of somnolence compared to standard doses (relative risk of 3.28; 95% confidence interval of 1.55–6.95; *p* = 0.002) [42]. Typically, a 2-week period is sufficient to assess the effects of antihistamine adjustments in CSU treatment [43]. Approximately 61% of CSU patients do not respond to standard licensed doses of sgAHs. Of these non-responders, only about 63% benefit from an increased dose [36]; the others can benefit from a therapeutic step up with regard to omalizumab.

### 2.2. Histamine Human Immunoglobulin

Histamine human immunoglobulin (histaglobulin, a combination of human normal immunoglobulin and histamine dihydrochloride that elicits the production of histamine-binding antibodies) is going to be studied in China. The study is currently not yet recruiting and will start in March 2025. The study should be closed by the end of 2025. A preliminary prospective study carried out in India found that the weekly subcutaneous administration of histaglobulin was able to reduce the UAS7 scores by >80% after 8 weeks and 45% of patients attained a complete remission without relevant side effects. [44]. A more recent case report confirmed these observations [45].

### 2.3. Leukotriene Receptor Antagonists

Leukotriene receptor antagonists (LRAs) block cysteinyl leukotrienes, which are potent pro-inflammatory mediators [46]. The potential role of LRAs in the treatment of CSU was described for the first time in 2000 in a case report [47]. This study reported that NSAID-induced exacerbations in a patient with CSU were successfully prevented with montelukast (10 mg once a day for 3 weeks) and zafirlukast (20 mg twice daily for 3 weeks) treatment.

The effectiveness of LRAs was further investigated one year later by the same group [48] in a 12-patient sample with steroid-dependent CSU. The team administered montelukast, 10 mg, once a day or zafirlukast, 20 mg, twice daily for 3 weeks. One patient was excluded because of intolerance (severe headache) and 6/11 patients reached the remission stage.

In a single-blind, placebo-controlled, and crossover clinical study in 2002, montelukast treatment outcomes were superior to placebo outcomes [49].

An Indian double-blind, randomized, and controlled trial published in 2017 [50] compared treatment with 10 mg of levocetirizine to treatment with a combination of 5 mg of levocetirizine and 10 mg of montelukast. Both therapies were effective in terms of disease control, but the addition of montelukast was useful in terms of reducing the dosage of cetirizine and its side effects, such as dizziness. Other studies reported the usefulness of montelukast as an added therapy [51,52].

In contrast to the studies mentioned, there is evidence that indicates the absence of an advantage of treating with LRAs compared to treating with antihistamines in a monotherapy setting [53,54].

Finally, a case report described a paradoxical exacerbation of CSU in a patient under treatment with antihistamines and montelukast [55].

In a systematic review considering 10 randomized controlled trials, no significant adverse effects or change in laboratory were observed in patients treated with LRAs [46].

### 2.4. Anti-Cytokine Therapies

#### 2.4.1. Canakinumab

Canakinumab is a human anti-IL-1 beta monoclonal antibody that is currently approved by the European Medicines Agency for treating periodic fever syndromes, Still’s disease, and gouty arthritis. In fact, IL-1beta plays a role in the pathogenesis of neutrophilic diseases. Moreover, many periodic fever syndromes can lead to cutaneous manifestations, such as urticaria. [56]. A phase II randomized double-blind placebo-controlled single-center study [57] investigated the efficacy of canakinumab compared to a placebo. Canakinumab was administered subcutaneously at a dosage of 150 mg once at baseline, but after 4 weeks, it was not superior to the placebo, suggesting the limited relevance of IL-1beta in the pathophysiology of CSU.

#### 2.4.2. Mepolizumab

Mepolizumab is a monoclonal antibody, targeting IL-5, that is approved for use in severe eosinophilic asthma, chronic rhinosinusitis with nasal polyposis, eosinophilic granulomatosis with polyangiitis, and hypereosinophilic syndrome. Since IL-5 appears to play an important role in CSU, mediating eosinophil migration to the skin during the period of active disease [58,59], this cytokine could be a new therapeutic target.

Two case reports, the first one including one patient and the second one, more recent, including three asthmatic patients with CSU, described the achievement of complete symptom resolution following mepolizumab treatment, with UCT scores reaching 15 after the first dose and symptom-free periods extending for up to six months [20,59]. Moreover, a phase I trial is currently underway to evaluate mepolizumab’s effectiveness in treating CSU (NCT03494881). There are still no data available on urticaria treatment with mepolizumab, which has shown a good safety profile for the treatment of other diseases.

#### 2.4.3. Reslizumab

Reslizumab is a monoclonal antibody targeting IL-5 approved for use in severe eosinophilic asthma. In a case report, it demonstrated significant effectiveness treating asthma, CSU, and cold urticaria [19].

#### 2.4.4. Secukinumab

Patients with CSU showed elevated serum levels of IL-17 and IL-23, which have been associated with disease activity, and a positive autologous serum skin test, an indicator of autoimmune CSU (aiCSU) [60]. Additionally, IL-17A expression was significantly higher in both lesional and non-lesional skin of CSU patients compared to the skin of healthy controls, where IL-17A expression was minimal or absent [61]. The preliminary findings indicated that all eight patients with antihistamine-resistant and omalizumab-resistant CSU who were treated with the anti-IL-17A mAb, secukinumab, experienced significant improvements in disease activity. In particular, the reduction in disease activity, assessed by UAS7, was 55% at 30 days and 82% at 90 days [61].

#### 2.4.5. Tildrakizumab

Since serum levels of IL-23 are increased in CSU patients, the anti-IL-23 mAb tildrakizumab has been administrated to treat patients with CSU refractory to omalizumab. Good control of the disease was obtained after four weeks in two out of three CSU patients. After 90 days, the overall reduction in disease activity from the baseline values ranged from 19% to 75% [62].

#### 2.4.6. Vixarelimab

Vixarelimab, a human mAb that targets the beta subunit of the oncostatin M receptor, inhibits the signaling pathways of IL-31 and oncostatin M, both of which contribute to pruritus. Vixarelimab has been successfully used in prurigo nodularis [63] and is currently undergoing a phase II trial to assess its efficacy in CSU (NCT03858634).

## 3. Inhibition of Mast Cell Activation

### 3.1. Anti IgE

#### 3.1.1. Omalizumab

##### Mechanism of Action

Omalizumab, a humanized monoclonal anti-IgE antibody, was initially developed to treat allergic respiratory disorders like asthma. It works by binding free IgE to the binding site of the high-affinity receptor for the fragment crystallizable region of immunoglobulin E (FcεRI) [43], preventing IgE from attaching to FcεRI on mast cells and halting the immunological cascade. In vitro experiments with basophils demonstrate that omalizumab is able to detach IgE from high-affinity IgE receptors [64]. Its potential for treating CSU was recognized in 2005 [65,66]. Phase II studies confirmed omalizumab’s efficacy for autoallergic CSU, particularly in patients with IgE autoantibodies to thyroperoxidase [67]. While the exact mechanism in CSU is not fully understood, omalizumab reduces free IgE levels and downregulates FcεRI expression in skin cells and basophils [68,69]. Other contributing mechanisms may include changes in mast cells, autoantibodies, coagulation abnormalities, and inflammatory cytokine levels [70,71]. Patients with type IIb autoimmunity, characterized by IgG and IgM antibodies that act against IgE receptors, experience more severe CSU and respond less effectively to omalizumab [72].

##### Clinical Response

The efficacy of omalizumab in treating CSU is supported by several phase III studies, including ASTERIA I, ASTERIA II, and GLACIAL [73,74,75]. These studies confirmed that omalizumab significantly outperformed placebos in terms of reducing urticaria activity and itch severity in patients aged 12–75 with moderate to severe CSU unresponsive to H1-antihistamines [76]. A meta-analysis of seven randomized controlled trials validated these findings, showing significant reductions in itch and wheal scores, particularly administering a 300 mg dosage every four weeks [77]. Additionally, the phase III trials highlighted that omalizumab increased the proportion of angioedema-free days [78].

Omalizumab is both well tolerated and effective across different patient populations, including children, adolescents, and older adults [79,80,81,82,83]. Despite CSU being more prevalent in females, both sexes respond similarly to omalizumab treatment, although relapses are more common in males [84]. The phase III POLARIS study [85] also demonstrated significant decreases in itch severity for omalizumab compared to placebos in patients affected by CSU with an inducible component [86]. Furthermore, there is limited information on omalizumab’s use in pregnant subjects with cancer or those undergoing treatment with other biological therapies [87,88].

Omalizumab significantly improves QoL, sleep, sexual function, anxiety, and work productivity in CSU patients, as demonstrated by patient-reported outcomes, which are assessed by UAS7 and the Urticaria Control Test (UCT) [89,90,91,92,93,94]. The phase IV SUNRISE and EXTEND-CIU studies show that disease control is achieved by week 12 (assessed by the UCT and UAS7), with sustained improvements in various patient-reported outcomes for 48 weeks [95,96,97].

Additionally, omalizumab benefits patients with CSU and angioedema, as evidenced by the X-ACT study [98,99]. However, more research is needed to assess its effectiveness treating isolated angioedema.

The response to omalizumab varies among patients, who can be classified into four categories: ‘early responders’ (ER), who experience a swift and comprehensive recovery within less than one month; ‘late responders’ (LR), who show complete improvement only after several months of therapy; ‘partial responders’ (PR), who exhibit some improvement but not a complete response; and ‘non-responders’ (NR), who do not respond at all [100,101,102]. Kaplan et al. [101] found a median time to complete response of 8–10 weeks with 300 mg of omalizumab, but responses occurred at times ranging from the first injection to up to 24 weeks. About half of the non-responders at week 12 responded by week 24. Early responders showed a rapid decrease in IgE and basophil FcεRI levels.

The treatment plans depend on the initial response. Non-responders and partial responders may need up-dosing and re-evaluation after three months, while good responders might benefit from lower dosing after 3–6 months [103]. Several studies found that up-dosing (to 450 mg or 600 mg, or at an increased frequency) is safe and effective in up to 60% of refractory patients, especially those with angioedema, basophil activation, high BMI values, prior cyclosporin treatment, greater ages, lower UCT scores, associate chronic inducible urticaria (CIndU), or lower IgE levels [104,105,106,107]. However, such an approach is not allowed in all countries by regulatory agencies.

A Korean group of researchers found that a low dose of omalizumab (150 mg/month) is effective at managing chronic spontaneous urticaria (CSU), offering a cost-effective option in settings where higher doses are not feasible due to financial constraints. Better responses to low-dose omalizumab treatment were observed in patients with mild disease activity, the absence of atopic comorbidities, and current smoking status [108].

About 50% of CSU patients achieve long-term remission (over 4 years) after one to two courses of omalizumab [109]. However, relapse can occur, particularly in those with high baseline UAS score or slow symptom decrease [110]. A retrospective cohort study conducted in Poland found that relapses commonly occurred within the first 6 weeks after discontinuing omalizumab treatment, with each additional point gained in terms of the UAS7 score increasing the risk of relapse by 5.4% [111]. Fortunately, re-treatment is as effective as the initial treatment, as shown in a study where 88% of patients re-treated after relapse regained control [112].

Furthermore, another therapeutic strategy is to extend the dosing interval in responders in which this seems to help with discontinuation [113].

##### Predictors of Response

The most reliable predictor of omalizumab response in CSU is the total IgE level [100,114,115,116,117,118,119,120]. Studies consistently show that non-responders typically have low baseline total IgE levels. These are typically lower than 40 IU/mL, and in some cases lower than 20 IU/mL. Early responders generally have IgE levels above 70 IU/mL. Ertas et al. [114,121] demonstrated that the best predictor of the response is the ratio of week 4 IgE levels to baseline IgE levels. Non-responders had a significantly lower ratio than partial and complete responders. This finding was supported by the work of Esteves Caldeira et al. [122]. Another predictor of a good response to omalizumab is the presence of elevated sFcεRI serum levels [115,123,124]. In a recent paper, Ji et al. found increased levels of Gal-9+ eosinophils and basophils in patients with high disease activity and a good response to omalizumab treatment. Omalizumab effectively reduces these levels in responders, suggesting that Gal-9+ cells may serve as predictors of treatment response [125].

Other response indicators include serum omalizumab trough levels [126], eosinopenia [127], antinuclear antibodies [128], and IL-31 [115,116].

A poor response was associated with basophil CD203c activity [129], higher anti-FcεRI IgG autoantibody levels [130], psychiatric disease, and thyroid antibodies [131]. Patients with poor or delayed responses to omalizumab often have a type IIb autoimmune form of CSU, characterized by IgG autoantibodies targeting IgE or its receptor (FcεRI) on mast cells. This leads to persistent cell activation and histamine release, which omalizumab may not adequately suppress, as its primary mechanism targets free IgE. [132,133].

Predictors of relapse after stopping omalizumab treatment include high IgE levels, high initial UAS7 scores, and an early response to treatment [110,114,116].

##### Safety

Omalizumab has demonstrated excellent safety over 20 years of various uses and dosages [134], as confirmed by both clinical trials and real-world studies [135]. A meta-analysis of 67 real-world studies indicated a 4% adverse event rate, matching the safety profile observed in clinical trials [136]. Nine-year long-term data showed no increase in side effects with extended omalizumab use [137,138]. Reports of anaphylaxis are very rare, with incidences between 0% and 0.09%, which are lower than the rates seen with most other biologics [139]. The US FDA reported more severe anaphylaxis cases in asthma patients than in those with urticaria [140,141].

The safety of omalizumab was further supported by a recent multinational cohort study that analyzed its long-term effectiveness and safety in treating chronic urticaria across 14 centers in 10 countries. The study involved 2325 patients and revealed an overall drug survival rate of 76% at 1 year, decreasing to 39% at 7 years. The primary reason for discontinuation was well-controlled disease (65%) [142]. However, a minority of patients discontinued treatment due to a lack of efficacy; in these cases, current guidelines suggest immunosuppressive therapy, with cyclosporin being the most commonly used.

#### 3.1.2. Ligelizumab

Ligelizumab is a second-generation anti-IgE monoclonal antibody, with 50 times higher affinity for IgE than omalizumab. It was developed for patients with CSU who were unresponsive to standard treatments. Initial studies showed that it provided rapid and effective relief with a long-lasting effect [143,144]. However, phase III trials (PEARL-1 and PEARL-2) were halted because ligelizumab, while superior to the placebo, did not significantly outperform omalizumab. The safety was consistent with that seen in previous studies [144].

#### 3.1.3. UB-221

UB-221 binds to IgE with high affinity, preventing IgE from interacting with the FcεRI receptor on mast cells and basophils. Additionally, UB-221 can bind to the IgE that is already bound to the CD23 receptor on B cells. This interaction promotes the CD23-mediated downregulation of IgE production. Unlike omalizumab, UB-221 can freely bind to CD23-bound IgE and form complexes with CD23, enhancing its ability to reduce IgE synthesis and the overall IgE levels in the body [22]. Based on the results of the phase I study (NCT03632291), UB-221 was well tolerated without serious adverse events. Currently, a phase II study is recruiting patients (NCT05298215).

#### 3.1.4. Miscellaneous Drugs Targeting IgE

Quilizumab, a monoclonal antibody targeting membrane-bound IgE, did not show any visible improvement in patients with CSU [145]. Another drug, UCB8600, was investigated for use treating CSU patients (NCT04444466), but the study was terminated by the company for reasons unrelated to safety. Furthermore, the IgE-Trap protein (YH35324), which has a high affinity for serum-free IgE, is currently being investigated in a phase I trial (NCT05960708) involving patients with chronic spontaneous urticaria (CSU) and cold urticaria.

### 3.2. Dupilumab

Dupilumab is a monoclonal antibody that blocks the alpha subunit of the interleukin-4 (IL-4) and interleukin-13 (IL-13) receptors, thereby inhibiting their signaling pathways. It is currently indicated for the treatment of type 2 inflammatory conditions such as atopic dermatitis, asthma, and chronic rhinosinusitis with nasal polyposis. Dupilumab’s efficacy in treating CSU was demonstrated in the placebo-controlled phase 3 clinical trial, LIBERTY-CSU CUPID Study A. This study investigated 138 biologic-naïve patients with CSU refractory to antihistamines and observed a significant reduction in UAS7, pruritus, and urticaria at week 24 [16]. These findings are also supported by a real-life study on 33 patients affected by CSU and treated with dupilumab [17]. However, another phase 3 study including 83 patients refractory to omalizumab, LIBERTY-CUPID Study B, was discontinued due to a lack of efficacy as it did not achieve statistical significance for the primary endpoints [16]. Additional papers have described a total of 25 patients with refractory CSU who responded positively to dupilumab [146,147,148,149,150,151,152,153,154,155,156,157,158,159].

Furthermore, by targeting B cells and reducing IgE levels, which in turn affects IgE receptor expression, dupilumab has shown potential in modifying the course of CSU. A case report indicated that 67% of patients maintained the remission of CSU for up to 22 months after discontinuing therapy with dupilumab [160]. These findings underscore the potential of dupilumab as a disease-modifying treatment in CSU [15]. The safety results were generally consistent with the known safety profile of dupilumab in its approved dermatological indications, with no differences with the placebo group [16].

### 3.3. Benralizumab

Benralizumab, a monoclonal antibody targeting the IL-5 receptor, has shown potential in treating CIndU, as evidenced by a case report where a patient with severe chronic symptomatic dermographism benefited from the treatment [21,161]. Although preliminary study results were promising, the drug demonstrated limited efficacy in a placebo-controlled randomized clinical trial, leading to the discontinuation of the development program (NCT04612725). Furthermore, a case report showed that in some cases, benralizumab can worsen CSU, although the exact mechanism behind this remains unclear [162].

### 3.4. Tezepelumab

Thymic stromal lymphopoietin (TSLP), an epithelial cell-derived cytokine, plays a critical role in initiating type 2 inflammation through both innate and adaptive immune pathways. Tezepelumab, an anti-TSLP mAb, has been shown to be safe, well tolerated, and effective in improving asthma control, also reducing the incidence of exacerbation and hospitalization in patients with severe asthma [163]. Increased levels of Th2-initiating cytokines, including TSLP, have been observed in the lesional skin of CSU patients [164]. A phase II trial of Tezepelumab for CSU has been completed (NCT04833855), with results pending publication.

### 3.5. MRGPRX2 Antagonists

Mas-related G protein-coupled receptor X2 (MRGPRX2) overexpression was observed in allergic and skin diseases, as CSU [165], and its downregulation in human or mice mast cells, leads to a reduction in mast cells degranulation [166]. MRGPRX2 is an important non-IgE-mediated pathway for mast cell activation and a potential therapeutic target for CSU [167,168]. Moreover, a recent study proposed that serum MRGPX2 may be a potential biomarker reflecting CSU activity, especially in naïve patients [169].

Some novel small molecules, designed through a computational approach and targeting MRGPRX2, were reported in a study published in 2023 [170]. In particular, the effects of the novel MRGPRX2 antagonists were assessed in vitro and in vivo using a mouse model of acute allergy and systemic anaphylaxis. It was observed that the small molecules inhibited both the early and the late phases of mast cell activation.

The therapeutic potential of MRGPRX2 antagonists was confirmed in a recent study [171] in which the molecules were tested on multiple functional assays in cell lines overexpressing human MRGPRX2, including isolated skin mast cells.

Two highly selective small molecule antagonists of MRGPRX2 are in trials for chronic spontaneous urticaria (EVO756, phase 1; EP262, phase 2 NCT06077773) and chronic inducible urticaria (EP262, phase 1b NCT06050928), but the results are still pending.

### 3.6. Complement Pathway Inhibitors

Another promising therapeutic target is the C5a/C5aR pathway. In CSU, the degranulation of mast cells by IgG autoantibodies requires them to bind to the IgE receptor and the activation of the classical complement cascade [172,173]. Another source of complement activation is the extrinsic coagulation pathway, which operates via the production of complement C5a, acting on the C5a receptor (C5aR) present on mast cells. Experimental studies have confirmed that the process may be inhibited by the C5aR antagonist, W-54011; however, studies in humans are still lacking to date [13,172].

## 4. Mast Cell Silencing

### Lirentelimab

Lirentelimab is a monoclonal antibody that targets the receptor of sialic acid-binding immunoglobulin-like lectin-8 (Siglec-8) on eosinophils and mast cells. Its effect leads to the depletion of eosinophils via apoptosis and the silencing of mast cells [173]. In preliminary clinical studies, lirentelimab showed improved disease control in both omalizumab-naïve and omalizumab-refractory patients with CSU, as well as in patients with CIndU. These improvements were assessed by the evaluation of the increases in UCT and UAS7 score [174]. However, in January 2024, the company announced that the primary endpoints of the phase II CSU trial were not met, and further development of the drug is unlikely [174,175].

## 5. Mast Cell Depletion

### 5.1. Barzolvolimab

The main regulators of mast cell biology are the tyrosine kinase receptor Kit (CD117), which is highly expressed by mast cells, and its ligand stem cell factor (SCF). The bond between the two molecules leads to the differentiation, chemotaxis, maturation, and survival of mast cells [176,177]. By preventing SCF from binding to Kit, the critical survival signals cease, and the mast cell undergoes apoptosis.

Barzolvolimab (CDX-0159) is a humanized immunoglobulin G1 kappa (IgG1κ) monoclonal antibody that binds to the extracellular domain of Kit with high specificity and sub-nanomolar affinity, preventing the activation by SCF [178].

It was demonstrated that mice deficient in either Kit or SCF [179] and patients under treatment with imatinib [180], a tyrosine kinase inhibitor, have significantly reductions in mast cell burden. Then, Alvarado et al. in 2022 demonstrated that barzolvolimab (CDX-0159) induces mast cell suppression [178].

A single-center, open-label phase 1b study (NCT04548869; EUDRA-CT 2020-002792-35 [18]) in 2022 presented preliminary results concerning the tolerability and the effectiveness of barzolvolimab in the treatment of chronic inducible urticaria. Adults between 18 and 75 years of age with a diagnosis of cold-induced urticaria or spontaneous dermographism for ≥3 months were included. A single dose of 3 mg/kg of barzolvolimab was administered intravenously on day 1 with a 12-week follow up. The drug exhibited a terminal half-life of 20.1  ±  7.1 days. No severe adverse event was detected; however, hair color changes (areas of hair lightening) and infusion-related reactions were often observed. All patients saw an improvement by week 12 in terms of disease control (UCT, TempTest^®^, FricTest^®^), quality of life (DLQI), serum parameters (tryptase, SCF) and histopathological features (mast cells in non-lesional skin). The trial is still ongoing, and the results will be available once the study is complete.

Two global phase 3 trials (randomized, double-blind, placebo-controlled) are still ongoing, investigating the efficacy, safety, and tolerability of barzolvolimab in adult participants with CSU (EMBARQ-CSU 1 and 2 [NCT06445023; NCT06455202]).

### 5.2. Briquilimab

Briquilimab (JSP191) is an unconjugated, aglycosilated anti-cKit monoclonal antibody that functionally blocks the interaction between cKit and SCF. The studies in phase 1b/2a SPOTLIGHT (NCT06353971) and BEACON (NCT06162728) are investigating the effect of subcutaneous Briquilimab in adults with cold urticaria and chronic spontaneous urticaria, respectively. Results are expected at the end of 2025.

## 6. Inhibition of Mast Cell Common Enzymatic Pathways

### 6.1. Bruton Tyrosine Kinase Inhibitors

Bruton tyrosine kinase (BTK) is a central player in the pathogenesis of CSU due to its critical role in FcεRI-mediated signaling, which is essential for mast cell and basophil activation. BTK facilitates the downstream signaling required for the activation and degranulation of these cells, leading to the release of inflammatory mediators like histamine, which contribute to CSU symptoms [26,181]. Additionally, BTK is involved in B-cell receptor (BCR) signaling, which is crucial to the production of autoantibodies by B cells [26].

By inhibiting BTK, it is possible to block both the FcεRI and BCR signaling pathways, thereby targeting the two key mechanisms driving CSU [26]. This finding makes BTK inhibitors promising therapeutic options for patients with antihistamine-refractory CSU.

BTK inhibitors (BTKis) are employed to treat several inflammatory and autoimmune conditions, as well as in cancer treatment [182]. Early-generation BTK inhibitors, like ibrutinib, were developed to treat B-cell malignancies but had limitations due to off-target effects and safety concerns (e.g., atrial fibrillation and bleeding) [26,183,184,185]. Newer BTK inhibitors, such as remibrutinib and fenebrutinib, are more selective, offering improved safety profiles while retaining efficacy [186]. Four oral BTKis—fenebrutinib, remibrutinib, rilzabrutinib, and TAS5315—have been or are currently being evaluated for use in chronic urticaria [26].

#### 6.1.1. Fenebrutinib

Fenebrutinib, a potent and highly selective reversible BTKi, has been shown to block IgE-mediated histamine release from mast cells in vitro [181]. In a recent double-blind, placebo-controlled phase II trial, fenebrutinib was effective in reducing disease activity in patients with sgAH-resistant CSU [187]. The drug was generally well tolerated, though some patients experienced reversible grade 2 to 3 alanine aminotransferase/aspartate transaminase (ALT/AST) abnormalities, particularly at higher doses. These findings were consistent across studies in rheumatoid arthritis and lupus patients as well [188,189]. The potential role of fenebrutinib in CSU was supported by Metz et al. [187]; however, the follow-up study (NCT03693625) was discontinued [190].

#### 6.1.2. Remibrutinib

Remibrutinib (LOU064), a novel, irreversible, and covalent BTKi, has demonstrated high selectivity and potency for BTK inhibition [26]. In phase II studies, remibrutinib showed good clinical efficacy and a favorable safety profile in patients with sgAHs-refractory CSU across a dose range of 10 to 200 mg daily [23]. The 25 mg twice-daily dose was particularly effective compared to placebos, reducing itching and hives as early as the first week of treatment, with effects sustained through week 12. Phase III trials confirmed these findings, meeting all primary and secondary endpoints and demonstrating rapid symptom control with a good safety profile [24,25]. Thus, remibrutinib appears to be a promising new treatment option for patients with sgAH-refractory CSU.

#### 6.1.3. Rilzabrutinib

Rilzabrutinib, a reversible, covalent, and selective BTKi, has shown efficacy and good tolerability in clinical trials for several autoimmune disorders, including chronic immune thrombocytopenia [26,191]. Its activity in CSU has been evaluated in a phase II trial (NCT05107115), although the results are not yet available.

#### 6.1.4. TAS5315

TAS5315, another highly potent and selective BTKi, has also been evaluated for treatment CSU in a phase II trial (NCT05335499), but, similarly, the results are still pending [26].

### 6.2. JAK-STAT Inhibitors

In the attempt to downregulate the expression of pro-inflammatory cytokines, some preliminary studies have considered employing JAK/STAT inhibitors in CSU treatment.

The Janus kinase–signal transducer and activator of transcription (JAK-STAT) is an intracellular pathway involved in the signaling of many inflammatory cytokines and other effector molecules. The JAK family of kinases includes JAK1, JAK2, JAK3, and tyrosine kinase 2 (Tyk2) [192]. JAK inhibitors are small molecules with anti-inflammatory and immunomodulatory properties that are currently employed to treat autoimmune and chronic inflammatory conditions [192]. Tofacitinib, a JAK1/3 inhibitor, caused a reduction in symptoms in four patients with CSU and was well tolerated [193]. Additionally, ruxolitinib, which inhibits JAK1/2, has also demonstrated efficacy in CSU treatment [194]. Ongoing investigations include TLL-018, a dual JAK1/Tyk2 inhibitor, and povorcitinib, a JAK1 inhibitor, both of which are being evaluated in phase I and II studies for CSU (NCT06396026, NCT05373355, and NCT05936567).

## 7. Miscellaneous Immunosuppressants

### 7.1. Corticosteroids

Systemic corticosteroids act as anti-inflammatory and immunomodulatory molecules. In particular, they diffuse passively across the cellular membrane and bind to the intracellular glucocorticoid receptor-creating complex, which is then translocated into the nucleus. This complex, binding to DNA sequences called glucocorticoid-responsive elements (GREs), blocks the promoter sites of pro-inflammatory genes (e.g., activator protein-1 and nuclear factor κB) [195] and those of the synthesis of cytokines [196]. On the other hand, it recruits sequences coding for anti-inflammatory molecules (e.g., lipocortin I and p11 and calpactin-binding protein) [197,198]. Moreover, glucocorticoids inhibit the secretion of inflammatory cytokines by affecting post-translational events [199].

According to the most recent European guidelines [1], systemic corticosteroids are a symptomatic short-term therapy, and they should not represent a first-line option. In particular, the appropriate dosage in adults should be between 20 and 50 mg/d of prednisone, the equivalent of up to 10 days of treatment. This regimen should be considered a rescue therapy for acute urticaria and should be used for acute exacerbations of CSU to reduce the disease duration/activity [200,201]. The main reasons for limiting therapy to the acute phase are the long-term side effects associated with corticosteroids, such as skin thinning, striae, hypertension, hirsutism, immunosuppression, hyperglycemia, osteoporosis, obesity, impaired wound healing, and mood disorders [197]. Moreover, there is a lack [202,203] of randomized controlled trials.

Finally, it should be mentioned that, according to evidence in the French literature, the administration of systemic corticosteroids for the treatment of CSU as first-line treatment induces resistance to anti-H1 and a rebound effect at each interruption [204,205].

### 7.2. Cyclosporin

Cyclosporin is a cyclic undecapeptide derived from a fungus, Tolypocladium inflatum, and is widely used as an anti-inflammatory and immunosuppressant. In particular, it acts by inhibiting the calcineurin/NFAT pathway and the JNK and p38 signaling pathway [206].

The vast majority of studies concerning the efficacy of cyclosporin in the treatment of CSU date back to the introduction of omalizumab in order to find a long-term treatment for patients who are refractory to antihistamines in monotherapy [207,208,209].

According to the most recent European urticaria guidelines [1], cyclosporin, with a dosage of 3.5–5 mg/kg per day [208,210], is reserved for patients who have not responded to high doses of SgAHs and omalizumab [211,212].

It was reported that cyclosporin, in association with cetirizine, is significantly more effective than placebos and cetirizine alone in reducing the severity of CSU after 8 weeks [213].

Cyclosporin is considered effective, and its safety is dose-dependent. It cannot be considered a first-line treatment because of its side effects, such as hypertension, nephrotoxicity, dyslipidemia, electrolytes alterations, hypertrichosis, higher rates of infection and neoplastic risk, and gingival hyperplasia [214].

In one study, patients that responded well to cyclosporine had high CRP levels before the treatment [215]; on the other hand, another study reveals that there are no positive predictive factors [210].

In 2020, Maoz Segal et al. [216] proposed the use of an intensified protocol with omalizumab plus an immune-suppressive agent to treat patients refractory to one of these drugs when used in monotherapy. The immunosuppressor most commonly combined with omalizumab in the protocol was cyclosporin. The authors concluded that an association protocol is safe and effective for recalcitrant CSU and may be indicated in patients with low baseline IgE levels.

### 7.3. Traditional Immunosuppressors/Immunomodulators Other than Cyclosporin

Although the evidence from publications is scarce, clinical experience suggests that traditional immunosuppressants other than cyclosporine, such as azathioprine, methotrexate, dapsone, hydroxychloroquine, and mycophenolate mofetil, may be useful in certain contexts.

Since omalizumab has drastically modified treatment paradigms for CSU, the use of traditional immunosuppressors is currently limited to cases refractory to multiple therapies. However, they still have an important role in developing countries due to their low cost.

#### 7.3.1. Azathioprine

Azathioprine acts as immunosuppressor through inhibition in intracellular purine synthesis, which results in decreased numbers of circulating B and T lymphocytes, reduced immunoglobulin synthesis, and diminished interleukin 2 (IL-2) secretion [217].

Azathioprine was found to be effective at a dosage of 150 mg/day in 2 patients [218] affected by CSU resistant to other immunosuppressors. Moreover, its efficacy, at a dosage of 50 mg/day, was confirmed in a single-blind randomized control trial in patients with autologous serum skin test-positive CSU [219]. Finally, in 2019, a randomized prospective study on 80 antihistamine-refractory patients concluded the non-inferiority of azathioprine compared to cyclosporin [220].

The most common side effects of azathioprine at the doses typically used in the treatment of rheumatic diseases include gastrointestinal intolerance and bone marrow suppression [217].

#### 7.3.2. Methotrexate

Methotrexate is an antimetabolite agent, highly similar to folic acid, that targets critical folate-dependent enzymatic steps in the de novo synthesis of purines and pyrimidines, resulting in a reduction in circulating leukocytes [221].

A systematic review published in 2022 [222] concluded that methotrexate may be a therapeutic agent in recalcitrant or steroid-dependent cases of CSU, but there is no evidence of its superiority to other first-line molecules, in particular antihistamines [223]. Methotrexate might be considered a further option in omalizumab-resistant patients [224] and can be used in combination with omalizumab itself [225]. Regarding what is reported in the literature, the maximal dose of methotrexate in CSU treatment is 25 mg/week [222].

The main side effects of methotrexate are gastrointestinal and hematologic toxicity, which can be controlled by a small amount of folic acid supplementation [226].

#### 7.3.3. Dapsone

Dapsone is an antibiotic that is effective in leprosy infection but also effective in skin inflammatory conditions interfering with the migration of neutrophils.

Dapsone has been described as effective [227,228,229] and superior to antihistamines in monotherapy [230]. In a double-blind placebo-controlled study, dapsone administration, at a dosage of 100 mg/day, saw superior results to placebos in patients that failed antihistamine treatment [231].

The most important side effects are hematologic; in particular, there is an augmented metahemoglobine level [232].

#### 7.3.4. Hydroxychloroquine

Hydroxychloroquine is an antimalaric drug commonly used as an immunomodulator and is anti-inflammatory when used in several autoimmune diseases [233].

In 2017, Boonpiyathad et al. [234]. conducted a randomized single-blinded placebo-controlled trial and an open-label comparison study to investigate the efficacy of hydroxychloroquine, administered at a dosage of 400 mg/day, in the treatment of antihistamine-refractory CSU. They reported the efficacy of hydroxychloroquine and its superiority to placebos and leukotriene receptor antagonist if added to the fourfold dose of H1-antihistamines. A real-world retrospective study in 2022 [235] investigated the effectiveness of the add-on therapy with omalizumab and hydroxychloroquine in patients refractory to antihistamines. The authors concluded that although omalizumab was superior, hydroxychloroquine was effective in two-thirds of treated patients and should be considered as a safe add-on option in monotherapy for CSU patients refractory to antihistamines.

The main adverse effect is retinopathy and for this reason patients treated with hydroxychloroquine should be periodically monitored [233].

#### 7.3.5. Mycophenolate Mofetil

Mycophenolate mofetil is a prodrug of mycophenolic acid which inhibits the enzyme inosine-5′-monophosphate dehydrogenase, leading to lymphocyte-selective immunosuppression [236].

The use of mycophenolate mofetil in CSU is mentioned in the literature by a few studies. In an open-label, uncontrolled trial [237], nine patients affected with CSU who were poorly responsive to antihistamines and/or corticosteroids were successfully treated with mycophenolate mophetil at a dosage of 1000 mg twice daily. The efficacy of mycophenolate mofetil (at the initial dose of 500 mg twice daily with progressive increments) was confirmed in another retrospective study in 2012 [238]. Although the adverse effect profile of MMF is comparatively benign, gastrointestinal adverse effects are a major concern [239].

#### 7.3.6. Rituximab

Rituximab is a chimeric murine/human monoclonal antibody against CD20 that has been investigated by a few authors for the treatment of selected recalcitrant CSU patients. The available data are contradictory. In some cases, rituximab was effective [240,241,242,243], while it was ineffective in others [244]. Moreover, in one case, rituximab triggered urticaria in a young patient treated for pemphigus vulgaris [245].

In conclusion, a high level of evidence concerning the use of rituximab in urticaria is lacking and it should be highlighted that its main side effect consists of long-term deep immunosuppression.

#### 7.3.7. Tranexamic Acid

Tranexamic acid is a synthetic derivative of lysine capable of inhibiting the conversion of plasminogen into plasmin. It is commonly used as an anti-hemorrhagic drug. The rationale for the use of tranexamic acid in the treatment of CSU comes from the evidence that the disease is often characterized by the activation of the coagulation cascade as well as fibrinolysis, with often high levels of D-dimer. A pilot study conducted on 68 patients in 2010 [246] reported that an elevated D-dimer level was associated with a more severe disease and with resistance to antihistamines. In this study, 5/8 cases with elevated D-dimer showed marked improvements regarding tranexamic acid in association with nadroparin.

The data concerning the correlation between D-dimer level, disease severity, and resistance to conventional therapies were confirmed by an Indian retrospective study in 2021 [247]. Nausea and diarrhea are the most common adverse events [248].

## 8. Conclusions

Chronic spontaneous urticaria (CSU) is a complex, long-lasting disease that significantly impacts on patients’ quality of life. In this review, we present a comprehensive overview of CSU treatments, including evidence on both established drugs and new promising molecules that have been the subject of preliminary studies. We also discuss therapies that, despite being linked to CSU’s pathomechanisms, have not demonstrated significant clinical outcomes.

Currently, omalizumab remains the best treatment option for most patients, representing a revolution that has dramatically improved disease outcomes and patients’ quality of life. No treatment currently surpasses omalizumab in terms of efficacy and tolerability. At present, only antihistamines and omalizumab are approved for the treatment of CSU, with no additional approved options available for patients resistant to omalizumab.

Traditional immunosuppressants and immunomodulators may be considered in cases where first-line therapy fails, either as alternatives or for use in combination with other treatments. However, these drugs tend to be less well tolerated compared to antihistamines and omalizumab.

The key pathways in chronic spontaneous urticaria are mast cell activation and degranulation, leading to the release of mediators like histamine, which causes itching, redness, and swelling. In addition to autoimmunity, autoallergy, the complement system, and coagulation, with the participation of other cells such as eosinophils, endothelial cells, basophils, B lymphocytes, T lymphocytes, and monocytes, mast cell activation potentially involves additional mechanisms. The role of epithelial-derived alarmins, which activate the group 2 innate lymphoid cells (ILC2 cells), promoting TH2 cytokines and allergen-specific IgE, thus triggering mast cell activation, is under investigation. Targets for current and future therapies include key receptors (FcεRI, C5aR, MRGPRX2), signaling pathways (BTK, SYK), and mediators (IL-4, IL-17, IL-31).

Among the molecules targeting various pathogenic pathways currently under study, the emerging drugs are represented by BTKis, like remibrutinib; monoclonal antibodies targeting IL-4 and IL-13, such as dupilumab; and anti-Kit agents, such as barzolvolimab, JAK inhibitors, and MRGPRX2 antagonists (Table 2 and Table 3). If these findings are confirmed, they may lead to the introduction of new disease-modifying drugs.

## Figures and Tables

**Table 1:** The first approach to the therapeutic treatment of chronic spontaneous urticaria (CSU).

Drug	Type of Molecule	Administration	Mechanism of Action	Response	
Second-generation H1-antihistamines (standard dose)	Second-generation H1-antihistamine	Oral, standard dosage (varies by drug)	Selective peripheral H1 receptor antagonist	Significant reduction in UAS7 score, fewer sedative effects compared to first-generation antihistamines	Step 1
		Oral, up to 4× standard dosage		Improved efficacy in non-responders to standard doses, increased risk of somnolence but still well-tolerated	Step 2
Omalizumab	Humanized monoclonal anti-IgE antibody	Subcutaneous, 300 mg every 4 weeks	Binds free IgE, preventing attachment of FcεRI to mast cells and basophils	Significant reduction in urticaria activity and angioedema, especially in patients unresponsive to antihistamines	Step 3
Cyclosporin	Immunosuppressant,	Oral, 3.5–5 mg/kg per day	Inhibits calcineurin/NFAT and JNK/p38 signaling pathways	Effective in patients unresponsive to antihistamines and omalizumab, dose-dependent safety profile	Step 4
Corticosteroids	Anti-inflammatory, immunosuppressant	Oral or intravenous, 20–50 mg/day, up to 10 days	Inhibits pro-inflammatory cytokines and immune response, binds glucocorticoid receptor	Short-term relief of acute exacerbations; not recommended for long-term use due to side effects such as hypertension, osteoporosis, and immunosuppression	Acute phase

According to European guidelines [1], the treatment of CSU begins with a standard dose of second-generation H1-antihistamines (step 1). If needed, the dose can be increased by up to fourfold (step 2). The third step involves the addition of omalizumab (step 3). When a good clinical response is achieved, a step-down approach can be considered. If the patient does not respond adequately, an immunosuppressant such as cyclosporine may be added (step 4). A short course of corticosteroids may be considered in the case of acute exacerbation.

**Table 2:** Breakthrough treatments for chronic spontaneous urticaria (CSU): monoclonal antibodies.

Drug	Target	Mechanism of Action	Administration	Response	Clinical Trial Phase	References
Dupilumab	IL-4 R á (IL-4; IL-13)	The block of the alpha subunit of the IL-4 receptor, which is shared with the IL-13 receptor, thereby inhibiting both IL-4 and IL-13 signaling pathways	Subcutaneous	A significant reduction in UAS7; showed efficacy in biologic-naïve patients but limited efficacy in omalizumab-refractory patients	Phase III	NCT04180488, (LIBERTY-CUPID CSU) [16,17]
Barzolvolimab (CDX-0159)	Kit	The inhibition of Kit receptor, reducing mast cell survival and activation	Intravenous	Although the phase III study is still ongoing, in phase II a reduction in activity and quality of life scores has been reported	Phase III	NCT06445023; NCT06455202 [18]
Tezepelumab	TSLP	The inhibition of TSLP, a key initiator of type 2 inflammation	Subcutaneous	Preliminary results indicate a good response; however, the final data of phase II study are still not available	Phase II	NCT04833855 [19]
Vixarelimab	IL-31 R	The block of the IL-31 and oncostatin M signaling pathways, reducing pruritus and inflammation	Subcutaneous	Although the phase II study in CSU is still ongoing, its promising results in the treatment of prurigo nodularis suggest potential benefits for CSU	Phase II	NCT03858634
Mepolizumab	IL-5	The inhibition of IL-5, reducing eosinophil migration and activation	Subcutaneous	Effective in reducing CSU symptoms, especially in patients with eosinophilic diseases	Phase I	NCT03494881 [20,21]
UB-221	IgE	Binding to IgE with high affinity, preventing interaction with mast cells and basophils	Intravenous	Although the phase II study in CSU is still ongoing, the drug appears to be promising due to its safety and its superior effectiveness in reducing IgE levels compared to omalizumab	Phase II	NCT05298215 [22]

IgE: immunoglobulin E; IL: interleukin; Kit: tyrosine kinase receptor Kit; R: receptor; TSLP: thymic stromal lymphopoietin; UAS7: Weekly Urticaria Activity Score.

**Table 3:** Breakthrough treatments for chronic spontaneous urticaria (CSU): small molecules.

Drug	Target	Mechanism of Action	Administration	Response	Clinical Trial Phase	References
Remibrutinib	BTK	The inhibition of BTK, a key component of FcεRI signaling in mast cells and basophils, thereby reducing the release of inflammatory mediators like histamine.	Oral	Rapid and sustained symptom control, good safety profile in antihistamine-refractory CSU	Phase III	NCT03926611, NCT05114057 [23,24,25]
Rilzabrutinib	BTK		Oral	Results of the phase II studies are still pending. However, given their mechanism of action, which closely mirrors that of remibrutinib, their potential effectiveness in CSU seems likely.	Phase II	NCT05107115
TAS 5315	BTK		Oral		Phase II	NCT05335499 [26]
TLL-018	JAK1	The selective inhibition of JAK1, reducing cytokine-driven inflammation.	Oral	Despite being in the early phase of study, its inhibitory effects on JAK 1 suggest it could be a promising therapeutic option for CSU.	Phase I	NCT06396026
Povorcitinib	JAK1, JAK2, TYK2	The inhibition of JAK1, JAK2, and TYK2, disrupting cytokine signaling.	Oral	Although the results of the phase I and II studies are still pending, their broad spectrum of action makes these molecules appear promising.	Phase II	NCT05373355 NCT05936567
EP 262	MRGPRX2	The block of MRGPRX2 (the non-IgE mast cell receptor)	Oral	Given the emerging role of MRGPRX2 in CSU pathophysiology, blocking this receptor makes the drug a highly promising candidate, although the results from the phase II trial are still pending.	Phase II	NCT06077773

BTK: Bruton tyrosine kinase; FcεRI: high-affinity receptor for the fragment crystallizable region of immunoglobulin E; JAK: Janus kinase; MRGPRX2: Mas-related G protein-coupled receptor X2; TYK: tyrosine kinase.

## References

[1] Zuberbier T., Abdul Latiff A.H., Abuzakouk M., Aquilina S., Asero R., Baker D., Ballmer-Weber B., Bangert C., Ben-Shoshan M., Bernstein J.A. (2022). The international EAACI/GA2LEN/EuroGuiDerm/APAAACI guideline for the definition, classification, diagnosis, and management of urticaria. Allergy.

[2] Maurer M., Weller K., Bindslev-Jensen C., Giménez-Arnau A., Bousquet P.J., Bousquet J., Canonica G.W., Church M.K., Godse K.V., Grattan C.E.H. (2011). Unmet clinical needs in chronic spontaneous urticaria. A GA2LEN task force report. Allergy.

[3] O’Donnell B.F., Lawlor F., Simpson J., Morgan M., Greaves M.W. (1997). The impact of chronic urticaria on the quality of life. Br. J. Dermatol..

[4] Church M.K., Kolkhir P., Metz M., Maurer M. (2018). The role and relevance of mast cells in urticaria. Immunol. Rev..

[5] Grattan C.E.H., Francis D.M., Hide M., Greaves M.W. (1991). Detection of circulating histamine releasing autoantibodies with functional properties of anti-IgE in chronic urticaria. Clin. Exp. Allergy.

[6] Hide M., Francis D.M., Grattan C., Hakimi J., Kochan J.P., Greaves M.W. (1993). Autoantibodies against the high-affinity IgE receptor as a cause of histamine release in chronic urticaria. N. Engl. J. Med..

[7] Altrichter S., Peter H.J., Pisarevskaja D., Metz M., Martus P., Maurer M. (2011). IgE mediated autoallergy against thyroid peroxidase—A novel pathomechanism of chronic spontaneous urticaria?. PLoS ONE.

[8] Schmetzer O., Lakin E., Topal F.A., Preusse P., Freier D., Church M.K., Maurer M. (2018). IL-24 is a common and specific autoantigen of IgE in patients with chronic spontaneous urticaria. J. Allergy Clin. Immunol..

[9] Asero R., Marzano A., Ferrucci S., Lorini M., Carbonelli V., Cugno M. (2020). Co-occurrence of IgE and IgG autoantibodies in patients with chronic spontaneous urticaria. Clin. Exp. Immunol..

[10] Asero R., Tedeschi A., Riboldi P., Cugno M. (2006). Plasma of patients with chronic urticaria shows signs of thrombin generation, and its intradermal injection causes wheal-and-flare reactions much more frequently than autologous serum. J. Allergy Clin. Immunol..

[11] Asero R., Tedeschi A., Coppola R., Griffini S., Paparella P., Riboldi P., Marzano A.V., Fanoni D., Cugno M. (2007). Activation of the tissue factor pathway of blood coagulation in patients with chronic urticaria. J. Allergy Clin. Immunol..

[12] Cugno M., Marzano A.V., Tedeschi A., Fanoni D., Venegoni L., Asero R. (2009). Expression of tissue factor by eosinophils in patients with chronic urticaria. Int. Arch. Allergy Immunol..

[13] Yanase Y., Matsuo Y., Takahagi S., Kawaguchi T., Uchida K., Ishii K., Tanaka A., Matsubara D., Ozawa K., Hide M. (2021). Coagulation factors induce human skin mast cell and basophil degranulation via activation of complement 5 and the C5a receptor. J. Allergy Clin. Immunol..

[14] Giménez-Arnau A.M., de Montjoye L., Asero R., Cugno M., Kulthanan K., Yanase Y., Hide M., Kaplan A.P. (2021). The Pathogenesis of Chronic Spontaneous Urticaria: The Role of Infiltrating Cells. J. Allergy Clin. Immunol. Pract..

[15] Maurer M., Kolkhir P., Pereira M.P., Siebenhaar F., Witte-Händel E., Bergmann K., Bonnekoh H., Buttgereit T., Fluhr J.W., Frischbutter S. (2024). Disease modification in chronic spontaneous urticaria. Allergy.

[16] Maurer M., Casale T.B., Saini S.S., Ben-Shoshan M., Giménez-Arnau A.M., Bernstein J.A., Yagami A., Stjepanovic A., Radin A., Staudinger H.W. (2024). Dupilumab in patients with chronic spontaneous urticaria (LIBERTY-CSU CUPID): Two randomized, double-blind, placebo-controlled, phase 3 trials. J. Allergy Clin. Immunol..

[17] Kudlaty E., Newell P., Chovatiya R. (2024). Dupilumab as Add-on Therapy for Management of Chronic Spontaneous Urticaria. J. Clin. Aesthet. Dermatol..

[18] Terhorst-Molawi D., Hawro T., Grekowitz E., Kiefer L., Merchant K., Alvarado D., Thomas L.J., Hawthorne T., Crowley E., Heath-Chiozzi M. (2023). Anti-KIT antibody, barzolvolimab, reduces skin mast cells and disease activity in chronic inducible urticaria. Allergy.

[19] Maurer M., Altrichter S., Metz M., Zuberbier T., Church M., Bergmann K.C. (2018). Benefit from reslizumab treatment in a patient with chronic spontaneous urticaria and cold urticaria. J. Eur. Acad. Dermatol. Venereol..

[20] Antonicelli L., Tontini C., Garritani M.S., Piga M.A., Bilò M.B. (2023). Efficacy of Mepolizumab in Patients With Severe Eosinophilic Asthma and Concomitant Severe Chronic Urticaria: An Example of Personalized Medicine?. J. Investig. Allergol. Clin. Immunol..

[21] Bergmann K.C., Altrichter S., Maurer M. (2019). Benefit of benralizumab treatment in a patient with chronic symptomatic dermographism. J. Eur. Acad. Dermatol. Venereol..

[22] Kuo B.S., Li C.H., Chen J.B., Shiung Y.Y., Chu C.Y., Lee C.H., Liu Y.J., Kuo J.H., Hsu C., Su H.W. (2022). IgE-neutralizing UB-221 mAb, distinct from omalizumab and ligelizumab, exhibits CD23-mediated IgE downregulation and relieves urticaria symptoms. J. Clin. Investig..

[23] Maurer M., Berger W., Giménez-Arnau A., Hayama K., Jain V., Reich A., Haemmerle S., Lheritier K., Walsh P., Xia S. (2022). Remibrutinib, a novel BTK inhibitor, demonstrates promising efficacy and safety in chronic spontaneous urticaria. J. Allergy Clin. Immunol..

[24] Novartis Novartis Phase III Data Confirm Sustained Efficacy and Long-Term Safety of Oral Remibrutinib in Chronic Spontaneous Urticaria. https://www.novartis.com/news/media-releases/novartis-phase-iii-data-confirm-sustained-efficacy-and-long-term-safety-oral-remibrutinib-chronic-spontaneous-urticaria.

[25] Jain V., Giménez-Arnau A., Hayama K., Reich A., Carr W., Tillinghast J., Dahale S., Lheritier K., Walsh P., Zharkov A. (2024). Remibrutinib demonstrates favorable safety profile and sustained efficacy in chronic spontaneous urticaria over 52 weeks. J. Allergy Clin. Immunol..

[26] Bernstein J.A., Maurer M., Saini S.S. (2024). BTK signaling—A crucial link in the pathophysiology of chronic spontaneous urticaria. J. Allergy Clin. Immunol..

[27] Xiang Y.-K., Fok J.S., Podder I., Yücel M.B., Özkoca D., Thomsen S.F., Kocatürk E. (2024). An update on the use of antihistamines in managing chronic urticaria. Expert. Opin. Pharmacother..

[28] Wang D., Guo Q., Wu Z., Li M., He B., Du Y., Zhang K., Tao Y. (2024). Molecular mechanism of antihistamines recognition and regulation of the histamine H1 receptor. Nat. Commun..

[29] He L., Yi W., Huang X., Long H., Lu Q. (2021). Chronic Urticaria: Advances in Understanding of the Disease and Clinical Management. Clin. Rev. Allergy Immunol..

[30] Sánchez J., Zakzuk J., Cardona R. (2016). Prediction of the Efficacy of Antihistamines in Chronic Spontaneous Urticaria Based on Initial Suppression of the Histamine-Induced Wheal. J. Investig. Allergol. Clin. Immunol..

[31] Recto M.T., Gabriel M.T., Kulthanan K., Tantilipikorn P., Aw D.C.-W., Lee T.H., Chwen C.C., Mutusamy S., Hao N.T., Quang V.T. (2017). Selecting optimal second-generation antihistamines for allergic rhinitis and urticaria in Asia. Clin. Mol. Allergy.

[32] Xiao X., Xue P., Shi Y., Yao J., Cao W., Zhang L., Zou Z., Zhou S., Wang C., Chen M. (2023). The efficacy and safety of high-dose nonsedating antihistamines in chronic spontaneous urticaria: A systematic review and meta-analysis of randomized clinical trials. BMC Pharmacol. Toxicol..

[33] Sinha V.V., Kalikar M.V., Mukhi J.I., Giradkar A.B., Sontakke S. (2023). Comparative study of efficacy and safety of cetirizine and bilastine in patients of chronic spontaneous urticaria: Open-label, randomized, parallel-group study. Perspect. Clin. Res..

[34] Shah B., Dhoot D., Choudhary A., Jangid N., Mistry D., Shah S., Kamat S., Barkate H. (2022). A Comparative, Three-Arm, Randomized Clinical Trial to Evaluate the Effectiveness and Tolerability of Bilastine vs Fexofenadine vs Levocetirizine at the Standard Dose and Bilastine vs Fexofenadine at Higher Than the Standard Dose (Up-Dosing) vs Levocetirizine and Hydroxyzine (in Combination) in Patients with Chronic Spontaneous Urticaria. Clin. Cosmet. Investig. Dermatol..

[35] Podder I., Das A., Ghosh S., Biswas D., Sengupta S., Chowdhury S.N. (2020). Effectiveness, safety, and tolerability of bilastine 20 mg vs levocetirizine 5 mg for the treatment of chronic spontaneous urticaria: A double-blind, parallel group, randomized controlled trial. Dermatol. Ther..

[36] Guillén-Aguinaga S., Presa I.J., Aguinaga-Ontoso E., Guillén-Grima F., Ferrer M. (2016). Updosing nonsedating antihistamines in patients with chronic spontaneous urticaria: A systematic review and meta-analysis. Br. J. Dermatol..

[37] Zuberbier T., Münzberger C., Haustein U., Trippas E., Burtin B., Mariz S., Henz B. (1996). Double-blind crossover study of high-dose cetirizine in cholinergic urticaria. Dermatology.

[38] Staevska M., Popov T.A., Kralimarkova T., Lazarova C., Kraeva S., Popova D., Church D.S., Dimitrov V., Church M.K. (2010). The effectiveness of levocetirizine and desloratadine in up to 4 times conventional doses in difficult-to-treat urticaria. J. Allergy Clin. Immunol..

[39] Siebenhaar F., Degener F., Zuberbier T., Martus P., Maurer M. (2009). High-dose desloratadine decreases wheal volume and improves cold provocation thresholds compared with standard-dose treatment in patients with acquired cold urticaria: A randomized, placebo-controlled, crossover study. J. Allergy Clin. Immunol..

[40] Giménez-Arnau A., Izquierdo I., Maurer M. (2009). The use of a responder analysis to identify clinically meaningful differences in chronic urticaria patients following placebo-controlled treatment with rupatadine 10 and 20 mg. J. Eur. Acad. Dermatol. Venereol..

[41] Cataldi M., Maurer M., Taglialatela M., Church M.K. (2019). Cardiac safety of second-generation H1-antihistamines when updosed in chronic spontaneous urticaria. Clin. Exp. Allergy.

[42] Zhou P., Zeng S., Fu L., Chen H., Li L. (2022). Efficacy and Safety of Intensive Nonsedating Antihistamines for Chronic Spontaneous Urticaria: A Meta-Analysis of Randomized Controlled Trials. Int. Arch. Allergy Immunol..

[43] Presta L.G., Lahr S.J., Shields R.L., Porter J.P., Gorman C.M., Fendly B.M., Jardieu P.M. (1993). Humanization of an antibody directed against IgE. J. Immunol..

[44] Rajesh G., Keerthi S., Karthikeyan K., Venkatesan M. (2016). Weekly injection of histaglobulin produces long-term remission in chronic urticaria: A prospective clinical study. Indian. J. Pharmacol..

[45] Kim H.S., Noh G. (2021). Induction of remission in 205 chronic urticaria by immunotherapy using immunoglobulin/histamine complex 206 (Histobulin™): A case report. Allergy Asthma Clin. Immunol..

[46] de Silva N.L., Damayanthi H., Rajapakse A.C., Rodrigo C., Rajapakse S. (2014). Leukotriene receptor antagonists for chronic urticaria: A systematic review. Allergy Asthma Clin. Immunol..

[47] Asero R. (2000). Leukotriene receptor antagonists may prevent NSAID-induced exacerbations in patients with chronic urticaria. Ann. Allergy Asthma Immunol..

[48] Asero R., Tedeschi A., Lorini M. (2001). Leukotriene receptor antagonists in chronic urticaria. Allergy.

[49] Erbagci Z. (2002). The leukotriene receptor antagonist montelukast in the treatment of chronic idiopathic urticaria: A single-blind, placebo-controlled, crossover clinical study. J. Allergy Clin. Immunol..

[50] Sarkar T.K., Sil A., Pal S., Ghosh C., Das N.K. (2017). Effectiveness and safety of levocetirizine 10 mg versus a combination of levocetirizine 5 mg and montelukast 10 mg in chronic urticaria resistant to levocetirizine 5 mg: A double-blind, randomized, controlled trial. Indian. J. Dermatol. Venereol. Leprol..

[51] Alkeraye S., AlRuhaimi D.K. (2021). The Addition of Montelukast for the Treatment of Chronic Idiopathic Urticaria. Cureus.

[52] Khan S., Lynch N. (2012). Efficacy of montelukast as added therapy in patients with chronic idiopathic urticaria. Inflamm. Allergy Drug Targets..

[53] Di Lorenzo G., Pacor M.L., Mansueto P., Esposito Pellitteri M., Lo Bianco C., Ditta V., Martinelli N., Rini G.B. (2004). Randomized placebo-controlled trial comparing desloratadine and montelukast in monotherapy and desloratadine plus montelukast in combined therapy for chronic idiopathic urticaria. J. Allergy Clin. Immunol..

[54] Godse K.V. (2006). Oral montelukast monotherapy is ineffective in chronic idiopathic urticaria: A comparison with oral cetirizine. Indian. J. Dermatol. Venereol. Leprol..

[55] Tedeschi A. (2009). Paradoxical exacerbation of chronic urticaria by H1-antihistamines and montelukast. Eur. Ann. Allergy Clin. Immunol..

[56] Haas N., Küster W., Zuberbier T., Henz B.M. (2004). Muckle-Wells syndrome: Clinical and histological skin findings compatible with cold air urticaria in a large kindred. Br. J. Dermatol..

[57] Maul J.T., Distler M., Kolios A., Maul L.V., Guillet C., Graf N., Imhof L., Lang C., Navarini A.A., Schmid-Grendelmeier P. (2021). Canakinumab Lacks Efficacy in Treating Adult Patients with Moderate to Severe Chronic Spontaneous Urticaria in a Phase II Randomized Double-Blind Placebo-Controlled Single-Center Study. J. Allergy Clin. Immunol. Pract..

[58] Antosz K., Batko J., Błażejewska M., Gawor A., Sleziak J., Gomułka K. (2024). Insight into IL-5 as a Potential Target for the Treatment of Allergic Diseases. Biomedicines.

[59] Magerl M., Terhorst D., Metz M., Altrichter S., Zuberbier T., Maurer M., Bergmann K.C. (2018). Benefit of mepolizumab treatment in a patient with chronic spontaneous urticaria. J. Dtsch. Dermatol. Ges..

[60] Atwa M.A., Emara A.S., Youssef N., Bayoumy N.M. (2014). Serum concentration of IL-17, IL-23 and TNF-α among patients with chronic spontaneous urticaria: Association with disease activity and autologous serum skin test. J. Eur. Acad. Dermatol. Venereol..

[61] Sabag D.A., Matanes L., Bejar J., Sheffer H., Barzilai A., Church M.K., Toubi E., Maurer M., Vadasz Z. (2020). Interleukin-17 is a potential player and treatment target in severe chronic spontaneous urticaria. Clin. Exp. Allergy.

[62] Bonnekoh H., Kiefer L., Buttgereit T., Kolkhir P., Lütke-Eversloh M., Scheffel J., Maurer M., Metz M. (2023). Anti-IL-23 treatment with tildrakizumab can be effective in omalizumab-refractory chronic spontaneous urticaria: A case series. J. Allergy Clin. Immunol. Pract..

[63] Sofen H., Bissonnette R., Yosipovitch G., Silverberg J.I., Tyring S., Loo W.J., Zook M., Lee M., Zou L., Jiang G.-L. (2023). Efficacy and safety of vixarelimab, a human monoclonal oncostatin M receptor β antibody, in moderate-to-severe prurigo nodularis: A randomised, double-blind, placebo-controlled, phase 2a study. EClinicalMedicine.

[64] Maggi L., Rossettini B., Montaini G., Matucci A., Vultaggio A., Mazzoni A., Palterer B., Parronchi P., Maggi E., Liotta F. (2018). Omalizumab dampens type 2 inflammation in a group of long-term treated asthma patients and detaches IgE from FcεRI. Eur. J. Immunol..

[65] Mankad V.S., Burks A.W. (2005). Omalizumab: Other indications and unanswered questions. Clin. Rev. Allergy Immunol..

[66] Kaplan A.P. (2004). Chronic urticaria: Pathogenesis and treatment. J. Allergy Clin. Immunol..

[67] Maurer M., Altrichter S., Bieber T., Biedermann T., Bräutigam M., Seyfried S., Brehler R., Grabbe J., Hunzelmann N., Jakob T. (2011). Efficacy and safety of omalizumab in patients with chronic urticaria who exhibit IgE against thyroperoxidase. J. Allergy Clin. Immunol..

[68] Metz M., Staubach P., Bauer A., Brehler R., Gericke J., Kangas M., Ashton-Chess J., Jarvis P., Georgiou P., Canvin J. (2017). Clinical efficacy of omalizumab in chronic spontaneous urticaria is associated with a reduction of FcεRI-positive cells in the skin. Theranostics.

[69] Kaplan A.P., Giménez-Arnau A.M., Saini S.S. (2017). Mechanisms of action that contribute to efficacy of omalizumab in chronic spontaneous urticaria. Allergy.

[70] Zhou B., Li J., Liu R., Zhu L., Peng C. (2022). The Role of Crosstalk of Immune Cells in Pathogenesis of Chronic Spontaneous Urticaria. Front. Immunol..

[71] Hoşgören-Tekin S., Eyüboğlu I.P., Akkiprik M., Giménez-Arnau A.M., Salman A. (2024). Inflammatory cytokine levels and changes during omalizumab treatment in chronic spontaneous urticaria. Arch. Dermatol. Res..

[72] Gericke J., Metz M., Ohanyan T., Weller K., Altrichter S., Skov P.S., Falkencrone S., Brand J., Kromminga A., Hawro T. (2017). Serum autoreactivity predicts time to response to omalizumab therapy in chronic spontaneous urticaria. J. Allergy Clin. Immunol..

[73] Kaplan A., Ledford D., Ashby M., Canvin J., Zazzali J.L., Conner E., Veith J., Kamath N., Staubach P., Jakob T. (2013). Omalizumab in patients with symptomatic chronic idiopathic/spontaneous urticaria despite standard combination therapy. J. Allergy Clin. Immunol..

[74] Maurer M., Rosén K., Hsieh H.-J., Saini S., Grattan C., Gimenéz-Arnau A., Agarwal S., Doyle R., Canvin J., Kaplan A. (2013). Omalizumab for the treatment of chronic idiopathic or spontaneous urticaria. N. Engl. J. Med..

[75] Saini S.S., Bindslev-Jensen C., Maurer M., Grob J.-J., Baskan E.B., Bradley M.S., Canvin J., Rahmaoui A., Georgiou P., Alpan O. (2015). Efficacy and safety of omalizumab in patients with chronic idiopathic/spontaneous urticaria who remain symptomatic on H1 antihistamines: A randomized, placebo-controlled study. J. Investig. Dermatol..

[76] Giménez-Arnau A.M. (2017). Omalizumab for treating chronic spontaneous urticaria: An expert review on efficacy and safety. Expert. Opin. Biol. Ther..

[77] Zhao Z.-T., Ji C.-M., Yu W.-J., Meng L., Hawro T., Wei J.-F., Maurer M. (2016). Omalizumab for the treatment of chronic spontaneous urticaria: A meta-analysis of randomized clinical trials. J. Allergy Clin. Immunol..

[78] Zazzali J.L., Kaplan A., Maurer M., Raimundo K., Trzaskoma B., Solari P.G., Antonova E., Mendelson M., Rosén K.E. (2016). Angioedema in the omalizumab chronic idiopathic/spontaneous urticaria pivotal studies. Ann. Allergy Asthma Immunol..

[79] Dekkers C., Aghdam M.A., Graaf M., Knulst A.C., Meijer Y., Reek J.M.P., Stadermann M.B., Röckmann H. (2021). Safety and effectiveness of omalizumab for the treatment of chronic urticaria in pediatric patients. Pediatr. Allergy Immunol..

[80] Ari A., Levy Y., Segal N., Maoz-Segal R., Benor S., Broides A., Horev A., Epstein-Rigbi N., Agmon-Levin N., Marcus N. (2020). Efficacy of omalizumab treatment for pediatric chronic spontaneous urticaria: A multi-center retrospective case series. Pediatr. Dermatol..

[81] Ocak M., Soyer O., Buyuktiryaki B., Sekerel B.E., Sahiner U.M. (2020). Omalizumab treatment in adolescents with chronic spontaneous urticaria: Efficacy and safety. Allergol. Immunopathol..

[82] Song X.T., Chen Y.D., Yu M., Liu B., Zhao Z.T., Maurer M. (2021). Omalizumab in children and adolescents with chronic urticaria: A 16-week real-world study. Allergy.

[83] Martina E., Damiani G., Grieco T., Foti C., Pigatto P.D.M., Offidani A. (2021). It is never too late to treat chronic spontaneous urticaria with omalizumab: Real-life data from a multicenter observational study focusing on elderly patients. Dermatol. Ther..

[84] Sirufo M.M., Bassino E.M., De Pietro F., Ginaldi L., De Martinis M. (2022). Sex differences in the efficacy of omalizumab in the treatment of chronic spontaneous urticaria. Int. J. Immunopathol. Pharmacol..

[85] Hide M., Park H.-S., Igarashi A., Ye Y.-M., Kim T.-B., Yagami A., Roh J., Lee J.-H., Chinuki Y., Youn S.W. (2017). Efficacy and safety of omalizumab in Japanese and Korean patients with refractory chronic spontaneous urticaria. J. Dermatol. Sci..

[86] Skander D., Allenova A., Maurer M., Kolkhir P. (2021). Omalizumab is effective in patients with chronic spontaneous urticaria plus multiple chronic inducible urticaria. Eur. Ann. Allergy Clin. Immunol..

[87] González-Medina M., Curto-Barredo L., Labrador-Horrillo M., Giménez-Arnau A. (2017). Omalizumab use during pregnancy for chronic spontaneous urticaria (CSU): Report of two cases. J. Eur. Acad. Dermatol. Venereol..

[88] Türk M., Carneiro-Leão L., Kolkhir P., Bonnekoh H., Buttgereit T., Maurer M. (2020). How to Treat Patients with Chronic Spontaneous Urticaria with Omalizumab: Questions and Answers. J. Allergy Clin. Immunol. Pract..

[89] Finlay A.Y., Kaplan A.P., Beck L.A., Antonova E.N., Balp M.M., Zazzali J., Khalil S., Maurer M. (2017). Omalizumab substantially improves dermatology-related quality of life in patients with chronic spontaneous urticaria. J. Eur. Acad. Dermatol. Venereol..

[90] Gimenéz-Arnau A.M., Spector S., Antonova E., Trzaskoma B., Rosén K., Omachi T.A., Stull D., Balp M.M., Murphy T. (2016). Improvement of sleep in patients with chronic idiopathic/spontaneous urticaria treated with omalizumab: Results of three randomized, double-blind, placebo-controlled studies. Clin. Transl. Allergy.

[91] Maurer M., Sofen H., Ortiz B., Kianifard F., Gabriel S., Bernstein J.A. (2017). Positive impact of omalizumab on angioedema and quality of life in patients with refractory chronic idiopathic/spontaneous urticaria: Analyses according to the presence or absence of angioedema. J. Eur. Acad. Dermatol. Venereol..

[92] Durmaz K., Ataseven A., Temiz S.A., Isik B., Dursun R. (2022). Does omalizumab use in chronic spontaneous urticaria results in improvement in sexual functions?. J. Cosmet. Dermatol..

[93] Porter E., Tierney E., Byrne B., Basheer-O’Dwyer S., Kanaan A., Ramsay B., Field S. (2022). “It has given me my life back”: A qualitative study exploring the lived experience of patients with chronic spontaneous urticaria on omalizumab. Clin. Exp. Dermatol..

[94] Tan M.G., Bailey A.M.J., Dorus B., Kirchhof M.G. (2024). Clinical Impacts of Omalizumab on the Psychiatric Comorbidities of Chronic Spontaneous Urticaria: A Systematic Review and Meta-Analysis. J. Drugs Dermatol..

[95] Bérard F., Ferrier Le Bouedec M.C., Bouillet L., Reguiai Z., Barbaud A., Cambazard F., Milpied B., Pelvet B., Kasujee I., Gharbi H. (2019). Omalizumab in patients with chronic spontaneous urticaria nonresponsive to H1-antihistamine treatment: Results of the phase IV open-label SUNRISE study. Br. J. Dermatol..

[96] Casale T.B., Win P.H., Bernstein J.A., Rosén K., Holden M., Iqbal A., Trzaskoma B.L., Yang M., Antonova E.N., Murphy T. (2018). Omalizumab response in patients with chronic idiopathic urticaria: Insights from the XTEND-CIU study. J. Am. Acad. Dermatol..

[97] Casale T.B., Murphy T.R., Holden M., Rajput Y., Yoo B., Bernstein J.A. (2019). Impact of omalizumab on patient-reported outcomes in chronic idiopathic urticaria: Results from a randomized study (XTEND-CIU). J. Allergy Clin. Immunol. Pract..

[98] Goswamy V.P., Lee K.E., McKernan E.M., Fichtinger P.S., Mathur S.K., Viswanathan R.K. (2022). Omalizumab for treatment of idiopathic angioedema. Ann. Allergy Asthma Immunol..

[99] Staubach P., Metz M., Chapman-Rothe N., Sieder C., Bräutigam M., Canvin J., Maurer M. (2016). Effect of omalizumab on angioedema in H1-antihistamine-resistant chronic spontaneous urticaria patients: Results from X-ACT, a randomized controlled trial. Allergy.

[100] Asero R., Ferrucci S.M., Calzari P., Consonni D., Cugno M. (2023). Thyroid Autoimmunity in CSU: A Potential Marker of Omalizumab Response?. Int. J. Mol. Sci..

[101] Kaplan A., Ferrer M., Bernstein J.A., Antonova E., Trzaskoma B., Raimundo K., Rosén K., Omachi T.A., Khalil S., Zazzali J.L. (2016). Timing and duration of omalizumab response in patients with chronic idiopathic/spontaneous urticaria. J. Allergy Clin. Immunol..

[102] Syrigos N., Grapsa D., Zande M., Tziotou M., Syrigou E. (2018). Treatment response to omalizumab in patients with refractory chronic spontaneous urticaria. Int. J. Dermatol..

[103] Giménez Arnau A.M., Valero Santiago A., Bartra Tomás J., Jáuregui Presa I., Labrador Horrillo M., Miquel Miquel F.J., Ortiz de Frutos J., Sastre J., Silvestre Salvador J.F., Ferrer Puga M. (2019). Therapeutic Strategy According to Differences in Response to Omalizumab in Patients with Chronic Spontaneous Urticaria. J. Investig. Allergol. Clin. Immunol..

[104] Metz M., Vadasz Z., Kocatürk E., Giménez-Arnau A.M. (2020). Omalizumab Updosing in Chronic Spontaneous Urticaria: An Overview of Real-World Evidence. Clin. Rev. Allergy Immunol..

[105] Brás R., Costa C., Limão R., Caldeira L.E., Paulino M., Pedro E. (2023). Omalizumab in Chronic Spontaneous Urticaria (CSU): Real-Life Experience in Dose/Interval Adjustments and Treatment Discontinuation. J. Allergy Clin. Immunol. Pract..

[106] Chen Q., Wang W., Yang X., Li S., Deng S., Wang H., Liu W., Ni B., Song Z. (2024). Characteristics and Clinical Significance of Atopy in Chronic Spontaneous Urticaria: A Cross-Sectional Observational Study. Int. Arch. Allergy Immunol..

[107] Pierrard G., Bernier C., Du-Thanh A., Bara C., Soria A., Castelain F., Boccon-Gibod I., Hacard F., Delaunay J., de Montjoye L. (2024). Characterization of omalizumab updosing patterns and predictive factors in chronic spontaneous urticaria: A prospective multicentric observational study. Allergy.

[108] Kim M.J., Kim B.R., Kim S.H., Chang Y.S., Youn S.W. (2023). Clinical Response to Low-dose Omalizumab Treatment in Chronic Spontaneous Urticaria: A Retrospective Study of 179 Patients. Acta Derm. Venereol..

[109] Di Bona D., Nettis E., Bilancia M., Ridolo E., Minenna E., Nizi M.C., Albanesi M., Caiaffa M.F., Macchia L. (2021). Duration of chronic spontaneous urticaria remission after omalizumab discontinuation: A long-term observational study. J. Allergy Clin. Immunol. Pract..

[110] Ferrer M., Giménez-Arnau A., Saldana D., Janssens N., Balp M.-M., Khalil S., Risson V. (2018). Predicting Chronic Spontaneous Urticaria Symptom Return After Omalizumab Treatment Discontinuation: Exploratory Analysis. J. Allergy Clin. Immunol. Pract..

[111] Kucharczyk A., Marczyk K., Kucharczyk B., Plisko R., Perkowska J., Owczarek W., Jahnz-Różyk K. (2024). Predicting relapse in chronic spontaneous urticaria: A retrospective cohort study evaluating omalizumab withdrawal regimens. Allergy.

[112] Sussman G., Hébert J., Gulliver W., Lynde C., Yang W.H., Papp K., Gooderham M., Chambenoit O., Khalil S., DeTakacsy F. (2020). Omalizumab Re-Treatment and Step-Up in Patients with Chronic Spontaneous Urticaria: OPTIMA Trial. J. Allergy Clin. Immunol. Pract..

[113] Salman A., Aktas M., Apti Sengun O. (2021). Remission of chronic spontaneous urticaria following omalizumab with gradually extended dosing intervals: Real-life data. Australas. J. Dermatol..

[114] Ertas R., Ozyurt K., Atasoy M., Hawro T., Maurer M. (2018). The clinical response to omalizumab in chronic spontaneous urticaria patients is linked to and predicted by IgE levels and their change. Allergy.

[115] Fok J.S., Kolkhir P., Church M.K., Maurer M. (2021). Predictors of treatment response in chronic spontaneous urticaria. Allergy.

[116] Marzano A.V., Genovese G., Casazza G., Fierro M.T., Dapavo P., Crimi N., Ferrucci S., Pepe P., Liberati S., Pigatto P.D. (2019). Predictors of response to omalizumab and relapse in chronic spontaneous urticaria: A study of 470 patients. J. Eur. Acad. Dermatol. Venereol..

[117] Straesser M.D., Oliver E., Palacios T., Kyin T., Patrie J., Borish L., Saini S.S., Lawrence M.G. (2018). Serum IgE as an immunological marker to predict response to omalizumab treatment in symptomatic chronic urticaria. J. Allergy Clin. Immunol. Pract..

[118] Weller K., Ohanyan T., Hawro T., Ellrich A., Sussman G., Koplowitz J., Gimenez-Arnau A.M., Peveling-Oberhag A., Staubach P., Metz M. (2018). Total IgE levels are linked to the response of chronic spontaneous urticaria patients to omalizumab. Allergy.

[119] Yu M., Terhorst-Molawi D., Altrichter S., Hawro T., Chen Y.D., Liu B., Song X.T., Zhao Z.T., Maurer M. (2021). Omalizumab in chronic inducible urticaria: A real-life study of efficacy, safety, predictors of treatment outcome and time to response. Clin. Exp. Allergy.

[120] Maurer M., Kolkhir P., Moñino-Romero S., Metz M. (2023). The Crucial Role of IgE as a Predictor of Treatment Response to Omalizumab in Chronic Spontaneous Urticaria. J. Allergy Clin. Immunol. Pract..

[121] Asero R. (2022). Clinical variables of severe chronic spontaneous urticaria from total IgE standpoint: A retrospective study. Eur. Ann. Allergy Clin. Immunol..

[122] Caldeira L.E., Bernardino A., Paulino M., Costa C. (2023). Four-week total IgE/baseline total IgE ratio: Biomarker for omalizumab good response in chronic spontaneous urticaria real-life patients. J. Allergy Clin. Immunol. Pract..

[123] Deza G., Bertolín-Colilla M., Sánchez S., Soto D., Pujol R.M., Gimeno R., Giménez-Arnau A.M. (2018). Basophil FcɛRI expression is linked to time to omalizumab response in chronic spontaneous urticaria. J. Allergy Clin. Immunol..

[124] Moñino-Romero S., Kolkhir P., Ohanyan T., Szépfalusi Z., Weller K., Metz M., Scheffel J., Maurer M., Altrichter S. (2024). Elevated baseline soluble FcεRI may be linked to early response to omalizumab treatment in chronic spontaneous urticaria. J. Eur. Acad. Dermatol. Venereol..

[125] Ji J., Tang M., Zhao Y., Zhang C., Shen Y., Zhou B., Liu C., Maurer M., Jiao Q. (2024). In chronic spontaneous urticaria, increased Galectin-9 expression on basophils and eosinophils is linked to high disease activity, endotype-specific markers, and response to omalizumab treatment. Allergy.

[126] Ghazanfar M.N., Bartko E.A., Arildsen N.S., Poulsen L.K., Jensen B.M., Enevold C., Holm J.G., Woetmann A., Ødum N., Thomsen S.F. (2022). Omalizumab serum levels predict treatment outcomes in patients with chronic spontaneous urticaria: A three-month prospective study. Clin. Exp. Allergy.

[127] Kolkhir P., Church M.K., Altrichter S., Skov P.S., Hawro T., Frischbutter S., Metz M., Maurer M. (2020). Eosinopenia, in Chronic Spontaneous Urticaria, Is Associated with High Disease Activity, Autoimmunity, and Poor Response to Treatment. J. Allergy Clin. Immunol. Pract..

[128] Ertaş R., Hawro T., Altrichter S., Özyurt K., Erol K., Ertaş K., Maurer M. (2020). Antinuclear antibodies are common and linked to poor response to omalizumab treatment in patients with CSU. Allergy.

[129] Palacios T., Stillman L., Borish L., Lawrence M. (2016). Lack of basophil CD203c-upregulating activity as an immunological marker to predict response to treatment with omalizumab in patients with symptomatic chronic urticaria. J. Allergy Clin. Immunol. Pract..

[130] Maronese C.A., Ferrucci S.M., Moltrasio C., Lorini M., Carbonelli V., Asero R., Marzano A.V., Cugno M. (2023). IgG and IgE Autoantibodies to IgE Receptors in Chronic Spontaneous Urticaria and Their Role in the Response to Omalizumab. J. Clin. Med..

[131] Cakmak M.E. (2022). Comparison of the Patients with Chronic Urticaria Who Responded and Did Not Respond to Omalizumab Treatment: A Single-Center Retrospective Study. Int. Arch. Allergy Immunol..

[132] Asero R., Marzano A.V., Cugno M. (2020). Unresponsiveness to Omalizumab in Chronic Spontaneous Urticaria. Curr. Treat. Options Allergy.

[133] Calzari P., Chiei Gallo A., Barei F., Bono E., Cugno M., Marzano A.V., Ferrucci S.M. (2024). Omalizumab for the Treatment of Chronic Spontaneous Urticaria in Adults and Adolescents: An Eight-Year Real-Life Experience. J. Clin. Med..

[134] Lowe P.J., Georgiou P., Canvin J. (2015). Revision of omalizumab dosing table for dosing every 4 instead of 2 weeks for specific ranges of bodyweight and baseline IgE. Regul. Toxicol. Pharmacol..

[135] Normansell R., Walker S., Milan S.J., Walters E.H., Nair P. (2014). Omalizumab for asthma in adults and children. Cochrane Database Syst. Rev..

[136] Tharp M.D., Bernstein J.A., Kavati A., Ortiz B., MacDonald K., Denhaerynck K., Abraham I., Lee C.S. (2019). Benefits and Harms of Omalizumab Treatment in Adolescent and Adult Patients with Chronic Idiopathic (Spontaneous) Urticaria: A Meta-analysis of “Real-world” Evidence. JAMA Dermatol..

[137] Di Bona D., Fiorino I., Taurino M., Frisenda F., Minenna E., Pasculli C., Kourtis G., Rucco A.S., Nico A., Albanesi M. (2017). Long-term “real-life” safety of omalizumab in patients with severe uncontrolled asthma: A nine-year study. Respir. Med..

[138] Harrison R.G., MacRae M., Karsh J., Santucci S., Yang W.H. (2015). Anaphylaxis and serum sickness in patients receiving omalizumab: Reviewing the data in light of clinical experience. Ann. Allergy Asthma Immunol..

[139] Gülsen A., Wedi B., Jappe U. (2020). Hypersensitivity reactions to biologics (part I): Allergy as an important differential diagnosis in complex immune-derived adverse events. Allergo J. Int..

[140] Li L., Wang Z., Cui L., Xu Y., Guan K., Zhao B. (2021). Anaphylactic risk related to omalizumab, benralizumab, reslizumab, mepolizumab, and dupilumab. Clin. Transl. Allergy.

[141] Casale T.B., Gimenez-Arnau A.M., Bernstein J.A., Holden M., Zuberbier T., Maurer M. (2023). Omalizumab for Patients with Chronic Spontaneous Urticaria: A Narrative Review of Current Status. Dermatol. Ther..

[142] Soegiharto R., Alizadeh Aghdam M., Sørensen J.A., van Lindonk E., Bulut Demir F., Porras N.M., Matsuo Y., Kiefer L., Knulst A.C., Maurer M. (2024). Multinational Drug Survival Study of Omalizumab in Patients with Chronic Urticaria and Potential Predictors for Discontinuation. JAMA Dermatol..

[143] Gasser P., Tarchevskaya S.S., Guntern P., Brigger D., Ruppli R., Zbären N., Kleinboelting S., Heusser C., Jardetzky T.S., Eggel A. (2020). The mechanistic and functional profile of the therapeutic anti-IgE antibody ligelizumab differs from omalizumab. Nat. Commun..

[144] Maurer M., Ensina L.F., Gimenez-Arnau A.M., Sussman G., Hide M., Saini S., Grattan C., Fomina D., Rigopoulos D., Berard F. (2024). Efficacy and safety of ligelizumab in adults and adolescents with chronic spontaneous urticaria: Results of two phase 3 randomised controlled trials. Lancet.

[145] Harris J.M., Cabanski C.R., Scheerens H., Samineni D., Bradley M.S., Cochran C., Staubach P., Metz M., Sussman G., Maurer M. (2016). A randomized trial of quilizumab in adults with refractory chronic spontaneous urticaria. J. Allergy Clin. Immunol..

[146] Lee J.K., Simpson R.S. (2019). Dupilumab as a novel therapy for difficult to treat chronic spontaneous urticaria. J. Allergy Clin. Immunol. Pract..

[147] Errichetti E., Stinco G. (2021). Recalcitrant chronic urticaria treated with dupilumab: Report of two instances refractory to H1-antihistamines, omalizumab and cyclosporine and brief literature review. Dermatol. Therapy..

[148] Sun Y., Lin S.Y., Lan C.E. (2022). Dupilumab as a rescue therapy for a chronic urticaria patient who showed secondary failure to omalizumab. Kaohsiung J. Med. Sci..

[149] Puxkandl V., Hoetzenecker W., Altrichter S. (2023). Case report: Severe chronic spontaneous urticaria successfully treated with omalizumab and dupilumab. Allergol. Select..

[150] Sirufo M.M., Catalogna A., Raggiunti M., De Pietro F., Ginaldi L., De Martinis M. (2022). Cholinergic Urticaria, an Effective and Safe “Off Label” Use of Dupilumab: A Case Report with Literature Review. Clin. Cosmet. Investig. Dermatol..

[151] Marchal V., Reguiai Z. (2023). Efficacity of dupilumab in severe idiopathic cold urticaria: A case report. J. Dermatol. Treat..

[152] Ferrucci S., Benzecry V., Berti E., Asero R. (2020). Rapid disappearance of both severe atopic dermatitis and cold urticaria following dupilumab treatment. Clin. Exp. Dermatol..

[153] Zhu C., Fok J.S., Lin L., Su H., Maurer M. (2022). Complete response to dupilumab in a patient with chronic spontaneous urticaria who did not tolerate omalizumab. JAAD Case Rep..

[154] Goodman B., Jariwala S. (2021). Dupilumab as a novel therapy to treat adrenergic urticaria. Ann. Allergy Asthma Immunol..

[155] Holm J.G., Sørensen J.A., Thomsen S.F. (2022). Concurrent use of omalizumab and dupilumab in a 47-year-old woman with chronic spontaneous urticaria and atopic dermatitis. Int. J. Dermatol..

[156] Föhr J., Herbst M., Jahn S. (2021). Treatment of simultaneously occurring urticaria and atopic dermatitis with dupilumab. Hautarzt.

[157] Valtellini L., Barei F., Zussino M., Marzano A.V., Ferrucci S.M. (2024). Dupilumab: A new frontier for chronic urticaria. A case series and review of the literature. Int. J. Dermatol..

[158] Feldborg S.E.B., Thomsen S.F., Vestergaard C. (2024). Treatment refractory chronic spontaneous urticaria may benefit from treatment with dupilumab: A case series of eight patients. J. Eur. Acad. Dermatol. Venereol..

[159] Altrichter S., Frischbutter S., Fok J.S., Kolkhir P., Jiao Q., Skov P.S., Metz M., Church M.K., Maurer M. (2020). The role of eosinophils in chronic spontaneous urticaria. J. Allergy Clin. Immunol..

[160] Abadeh A., Lee J.K. (2022). Long-term follow-up of patients treated with dupilumab for chronic spontaneous urticaria: A case report. SAGE Open Med. Case Rep..

[161] Bernstein J.A., Singh U., Rao M.B., Berendts K., Zhang X., Mutasim D. (2020). Benralizumab for Chronic Spontaneous Urticaria. N. Engl. J. Med..

[162] Magen E., Komarova I., Magen I., Phirtskhalava S. (2022). Case of benralizumab-induced exacerbations of chronic spontaneous urticaria. Clin. Case Rep..

[163] Menzies-Gow A., Corren J., Bourdin A., Chupp G., Israel E., Wechsler M.E., Brightling C.E., Griffiths J.M., Hellqvist Å., Bowen K. (2021). Tezepelumab in Adults and Adolescents with Severe, Uncontrolled Asthma. N. Engl. J. Med..

[164] Kay A.B., Clark P., Maurer M., Ying S. (2015). Elevations in T-helper-2-initiating cytokines (interleukin-33, interleukin-25 and thymic stromal lymphopoietin) in lesional skin from chronic spontaneous (‘idiopathic’) urticaria. Br. J. Dermatol..

[165] Shtessel M., Limjunyawong N., Oliver E.T., Chichester K., Gao L., Dong X., Saini S.S. (2021). MRGPRX2 Activation Causes Increased Skin Reactivity in Patients with Chronic Spontaneous Urticaria. J. Investig. Dermatol..

[166] McNeil B.D., Pundir P., Meeker S., Han L., Undem B.J., Kulka M., Dong X. (2015). Identification of a mast-cell-specific receptor crucial for pseudo-allergic drug reactions. Nature.

[167] Ogasawara H., Noguchi M. (2021). Therapeutic Potential of MRGPRX2 Inhibitors on Mast Cells. Cells.

[168] Lerner L., Babina M., Zuberbier T., Stevanovic K. (2024). Beyond Allergies-Updates on The Role of Mas-Related G-Protein-Coupled Receptor X2 in Chronic Urticaria and Atopic Dermatitis. Cells.

[169] Lao K., Mak H.W.F., Chiang V., Kumar M., Chow B.K.C., Li P.H. (2024). Mas-Related G-Protein Coupled Receptor-X2 and Chemokine (C-C Motif) Ligand 2 Correlate with Disease Activity Among Treatment-Naïve Chinese Patients with Chronic Spontaneous Urticaria. Clin. Exp. Allergy.

[170] Kumar M., Duraisamy K., Annapureddy R.R., Chan C.B., Chow B.K. (2023). Novel small molecule MRGPRX2 antagonists inhibit a murine model of allergic reaction. J. Allergy Clin. Immunol..

[171] Wollam J., Solomon M., Villescaz C., Lanier M., Evans S., Bacon C., Freeman D., Vasquez A., Vest A., Napora J. (2024). Inhibition of mast cell degranulation by novel small molecule MRGPRX2 antagonists. J. Allergy Clin. Immunol..

[172] Ferrer M., Nakazawa K., Kaplan A.P. (1999). Complement dependence of histamine release in chronic urticaria. J. Allergy Clin. Immunol..

[173] Kolkhir P., Giménez-Arnau A.M., Kulthanan K., Peter J., Metz M., Maurer M. (2022). Urticaria. Nat. Rev. Dis. Primers..

[174] Altrichter S., Staubach P., Pasha M., Singh B., Chang A.T., Bernstein J.A., Rasmussen H.S., Siebenhaar F., Maurer M. (2022). An open-label, proof-of-concept study of lirentelimab for antihistamine-resistant chronic spontaneous and inducible urticaria. J. Allergy Clin. Immunol..

[175] Muñoz M., Kocatürk E., Maurer M., Kolkhir P. (2024). Emerging Therapeutics in Chronic Urticaria. Immunol. Allergy Clin. N. Am..

[176] Valent P., Akin C., Hartmann K., Nilsson G., Reiter A., Hermine O., Sotlar K., Sperr W.R., Escribano L., George T.I. (2020). Mast cells as a unique hematopoietic lineage and cell system: From Paul Ehrlich’s visions to precision medicine concepts. Theranostics.

[177] Davis M.I., Hunt J.P., Herrgard S., Ciceri P., Wodicka L.M., Pallares G., Hocker M., Treiber D.K., Zarrinkar P.P. (2011). Comprehensive analysis of kinase inhibitor selectivity. Nat. Biotechnol..

[178] Alvarado D., Maurer M., Gedrich R., Seibel S.B., Murphy M.B., Crew L., Goldstein J., Crocker A., Vitale L.A., Morani P.A. (2022). Anti-KIT monoclonal antibody CDX-0159 induces profound and durable mast cell suppression in a healthy volunteer study. Allergy.

[179] Grimbaldeston M.A., Chen C.-C., Piliponsky A.M., Tsai M., Tam S.-Y., Galli S.J. (2005). Mast cell-deficient W-sash c-kit mutant Kit W-sh/W-sh mice as a model for investigating mast cell biology in vivo. Am. J. Pathol..

[180] Cerny-Reiterer S., Rabenhorst A., Stefanzl G., Herndlhofer S., Hoermann G., Müllauer L., Baumgartner S., Beham-Schmid C., Sperr W.R., Mannhalter C. (2014). Long-term treatment with imatinib results in profound mast cell deficiency in Ph+ chronic myeloid leukemia. Oncotarget.

[181] Hata D., Kawakami Y., Inagaki N., Lantz C.S., Kitamura T., Khan W.N., Maeda-Yamamoto M., Miura T., Han W., Hartman S.E. (1998). Involvement of Bruton’s tyrosine kinase in FcepsilonRI-dependent mast cell degranulation and cytokine production. J. Exp. Med..

[182] Shen P., Wang Y., Jia X., Xu P., Qin L., Feng X., Li Z., Qiu Z. (2022). Dual-target Janus kinase (JAK) inhibitors: Comprehensive review on the JAK-based strategies for treating solid or hematological malignancies and immune-related diseases. Eur. J. Med. Chem..

[183] Zarrin A.A., Bao K., Lupardus P., Vucic D. (2021). Kinase inhibition in autoimmunity and inflammation. Nat. Rev. Drug Discov..

[184] Gu D., Tang H., Wu J., Li J., Miao Y. (2021). Targeting Bruton tyrosine kinase using non-covalent inhibitors in B cell malignancies. J. Hematol. Oncol..

[185] Estupiñán H.Y., Berglöf A., Zain R., Smith C.I.E. (2021). Comparative Analysis of BTK Inhibitors and Mechanisms Underlying Adverse Effects. Front. Cell Dev. Biol..

[186] Gabizon R., London N. (2020). A Fast and Clean BTK Inhibitor. J. Med. Chem..

[187] Metz M., Sussman G., Gagnon R., Staubach P., Tanus T., Yang W.H., Lim J.J., Clarke H.J., Galanter J., Chinn L.W. (2021). Fenebrutinib in H1 antihistamine-refractory chronic spontaneous urticaria: A randomized phase 2 trial. Nat. Med..

[188] Cohen S., Tuckwell K., Katsumoto T.R., Zhao R., Galanter J., Lee C., Rae J., Toth B., Ramamoorthi N., Hackney J.A. (2020). Fenebrutinib versus Placebo or Adalimumab in Rheumatoid Arthritis: A Randomized, Double-Blind, Phase II Trial (ANDES Study). Arthritis Rheumatol..

[189] Isenberg D., Furie R., Jones N.S., Guibord P., Galanter J., Lee C., McGregor A., Toth B., Rae J., Hwang O. (2021). Efficacy, Safety, and Pharmacodynamic Effects of the Bruton’s Tyrosine Kinase Inhibitor Fenebrutinib (GDC-0853) in Systemic Lupus Erythematosus: Results of a Phase II, Randomized, Double-Blind, Placebo-Controlled Trial. Arthritis Rheumatol..

[190] Asero R., Ferrucci S., Tedeschi A., Cugno M. (2022). Biologics for chronic spontaneous urticaria: Toward a personalized treatment. Expert. Rev. Clin. Immunol..

[191] Murakami J., Senoo Y., Tanimoto T. (2022). Rilzabrutinib in Immune Thrombocytopenia. N. Engl. J. Med..

[192] Damsky W., King B.A. (2017). JAK inhibitors in dermatology: The promise of a new drug class. J. Am. Acad. Dermatol..

[193] Mansouri P., Mozafari N., Chalangari R., Martits-Chalangari K. (2022). Efficacy of oral tofacitinib in refractory chronic spontaneous urticaria and urticarial vasculitis. Dermatol. Ther..

[194] Fukunaga A., Ito M., Nishigori C. (2018). Efficacy of Oral Ruxolitinib in a Patient with Refractory Chronic Spontaneous Urticaria. Acta Derm. Venereol..

[195] Auphan N., DiDonato J.A., Rosette C., Helmberg A., Karin M. (1995). Immunosuppression by glucocorticoids: Inhibition of NF-kappa B activity through induction of I kappa B synthesis. Science.

[196] Almawi W.Y., Beyhum H.N., Rahme A.A., Rieder M.J. (1996). Regulation of cytokine and cytokine receptor expression by glucocorticoids. J. Leukoc. Biol..

[197] Cain D.W., Cidlowski J.A. (2017). Immune regulation by glucocorticoids. Nat. Rev. Immunol..

[198] Brazzini B., Pimpinelli N. (2002). New and established topical corticosteroids in dermatology: Clinical pharmacology and therapeutic use. Am. J. Clin. Dermatol..

[199] Rhen T., Cidlowski J.A. (2005). Antiinflammatory action of glucocorticoids—New mechanisms for old drugs. N. Engl. J. Med..

[200] Asero R., Tedeschi A. (2010). Usefulness of a short course of oral prednisone in antihistamine-resistant chronic urticaria: A retrospective analysis. J. Investig. Allergol. Clin. Immunol..

[201] Zuberbier T., Iffländer J., Semmler C., Henz B.M. (1996). Acute urticaria: Clinical aspects and therapeutic responsiveness. Acta Derm. Venereol..

[202] Yen H., Yen H., Huang C.H., Huang I.H., Hung W.K., Su H.J., Tai C.C., Haw W.W.Y., Flohr C., You Z.Z.N. (2023). Systematic Review and Critical Appraisal of Urticaria Clinical Practice Guidelines: A Global Guidelines in Dermatology Mapping Project (GUIDEMAP). J. Allergy Clin. Immunol. Pract..

[203] Chu X., Wang J., Ologundudu L., Brignardello-Petersen R., Guyatt G.H., Oykhman P., Bernstein J.A., Saini S.S., Beck L.A., Waserman S. (2024). Efficacy and Safety of Systemic Corticosteroids for Urticaria: A Systematic Review and Meta-Analysis of Randomized Clinical Trials. J. Allergy Clin. Immunol. Pract..

[204] Augey F., Guillot-Pouget I., Gunera-Saad N., Berard F., Nicolas J.F. (2008). Impact of corticosteroid withdrawal in chronic urticaria: A prospective study of 17 patients. Ann. Dermatol. Venereol..

[205] Augey F., Nosbaum A., Ben-Said B., Bérard F., Nicolas J.F. (2011). Chronic urticaria and corticodependence: Corticosteroids have no role in the treatment of urticaria. Ann. Dermatol. Venereol..

[206] Lee H., Myoung H., Kim S.M. (2023). Review of two immunosuppressants: Tacrolimus and cyclosporine. J. Korean Assoc. Oral. Maxillofac. Surg..

[207] Grattan C.E., O’Donnell B.F., Francis D.M., Niimi N., Barlow R.J., Seed P.T., Kobza Black A., Greaves M.W. (2000). Randomized double-blind study of cyclosporin in chronic “idiopathic” urticaria. Br. J. Dermatol..

[208] Di Leo E., Nettis E., Aloia A., Moschetta M., Carbonara M., Dammacco F., Vacca A. (2011). Cyclosporin-A efficacy in chronic idiopathic urticaria. Int. J. Immunopathol. Pharmacol..

[209] Loria M.P., Dambra P.P., D’Oronzio L., Nettis E., Pannofino A., Cavallo E., Ferrannini A., Tursi A. (2001). Cyclosporin A in patients affected by chronic idiopathic urticaria: A therapeutic alternative. Immunopharmacol. Immunotoxicol..

[210] Kulthanan K., Chaweekulrat P., Komoltri C., Hunnangkul S., Tuchinda P., Chularojanamontri L., Maurer M. (2018). Cyclosporine for Chronic Spontaneous Urticaria: A Meta-Analysis and Systematic Review. J. Allergy Clin. Immunol. Pract..

[211] LaCava A.F., Fadugba O.O. (2023). Cyclosporine for omalizumab-refractory chronic urticaria: A report of five cases. Allergy Asthma Clin. Immunol..

[212] Mateu-Arrom L., Giménez-Arnau A.M., Expósito-Serrano V., Bonfill-Ortí M., Serra-Baldrich E., Yélamos O., Spertino J. (2024). Cyclosporine for the treatment of chronic spontaneous urticaria refractory to antihistamines and omalizumab: A case series. Int. J. Dermatol..

[213] Vena G.A., Cassano N., Colombo D., Peruzzi E., Pigatto P. (2006). Neo-I-30 Study Group. Cyclosporine in chronic idiopathic urticaria: A double-blind, randomized, placebo-controlled trial. J. Am. Acad. Dermatol..

[214] Tapia C., Nessel T.A., Zito P.M. (2024). Cyclosporine. StatPearls [Internet].

[215] Kocatürk E., Başkan E.B., Küçük Ö.S., Özdemir M., Örnek S., Can P.K., Haşal E., Engin B., Atakan N., Alpsoy E. (2022). Omalizumab versus cyclosporin-A for the treatment of chronic spontaneous urticaria: Can we define better-responding endotypes?. An. Bras. Dermatol..

[216] Maoz-Segal R., Levy T., Haj-Yahia S., Offengenden I., Iancovich-Kidon M., Agmon-Levin N. (2020). Combination therapy with omalizumab and an immune-suppressive agent for resistant chronic spontaneous rrticaria—A real-life experience. World Allergy Organ J..

[217] Pizzorno G., Diasio R.B., Cheng Y.C. (2003). Purine Analogs. Holland-Frei Cancer Medicine [Internet].

[218] Tal Y., Toker O., Agmon-Levin N., Shalit M. (2015). Azathioprine as a therapeutic alternative for refractory chronic urticaria. Int. J. Dermatol..

[219] Ghoshal L., Bhanja D.C., Das S., Das S., Roy A.K. (2015). Azathioprine in autologous serum skin test positive chronic urticaria: A case-control study in a tertiary care hospital of eastern India. Indian Dermatol. Online J..

[220] Pathania Y.S., Bishnoi A., Parsad D., Kumar A., Kumaran M.S. (2019). Comparing azathioprine with cyclosporine in the treatment of antihistamine refractory chronic spontaneous urticaria: A randomized prospective active-controlled non-inferiority study. World Allergy Organ J..

[221] Cronstein B.N., Aune T.M. (2020). Methotrexate and its mechanisms of action in inflammatory arthritis. Nat. Rev. Rheumatol..

[222] Sandhu J., Kumar A., Gupta S.K. (2022). The therapeutic role of methotrexate in chronic urticaria: A systematic review. Indian. J. Dermatol. Venereol. Leprol..

[223] Sharma V.K., Singh S., Ramam M., Kumawat M., Kumar R. (2014). A randomized placebo-controlled double-blind pilot study of methotrexate in the treatment of H1 antihistamine-resistant chronic spontaneous urticaria. Indian. J. Dermatol. Venereol. Leprol..

[224] Unsel M. (2021). Safety of Methotrexate in Chronic Urticaria Unresponsive to Omalizumab. Iran. J. Allergy Asthma Immunol..

[225] Garbayo-Salmons P., Butt S., Dawe R.S. (2021). Methotrexate combined with omalizumab for difficult to treat urticaria: A further step-up treatment?. Clin. Exp. Dermatol..

[226] Shea B., Swinden M.V., Ghogomu E.T., Ortiz Z., Katchamart W., Rader T., Bombardier C., Wells G.A., Tugwell P. (2014). Folic acid and folinic acid for reducing side effects in patients receiving methotrexate for rheumatoid arthritis. J. Rheumatol..

[227] Cassano N., D’Argento V., Filotico R., Vena G.A. (2005). Low-dose dapsone in chronic idiopathic urticaria: Preliminary results of an open study. Acta Derm. Venereol..

[228] Noda S., Asano Y., Sato S. (2012). Long-term complete resolution of severe chronic idiopathic urticaria after dapsone treatment. J. Dermatol..

[229] Liang S.E., Hoffmann R., Peterson E., Soter N.A. (2019). Use of Dapsone in the Treatment of Chronic Idiopathic and Autoimmune Urticaria. JAMA Dermatol..

[230] Engin B., Ozdemir M. (2008). Prospective randomized non-blinded clinical trial on the use of dapsone plus antihistamine vs. antihistamine in patients with chronic idiopathic urticaria. J. Eur. Acad. Dermatol. Venereol..

[231] Morgan M., Cooke A., Rogers L., Adams-Huet B., Khan D.A. (2014). Double-blind placebo-controlled trial of dapsone in antihistamine refractory chronic idiopathic urticaria. J. Allergy Clin. Immunol. Pract..

[232] Kurien G., Jamil R.T., Preuss C.V. (2024). Dapsone. StatPearls [Internet].

[233] Schrezenmeier E., Dörner T. (2020). Mechanisms of action of hydroxychloroquine and chloroquine: Implications for rheumatology. Nat. Rev. Rheumatol..

[234] Boonpiyathad T., Sangasapaviliya A. (2017). Hydroxychloroquine in the treatment of anti-histamine refractory chronic spontaneous urticaria, randomized single-blinded placebo-controlled trial and an open label comparison study. Eur. Ann. Allergy Clin. Immunol..

[235] Khan N., Epstein T.E., DuBuske I., Strobel M., Bernstein D.I. (2022). Effectiveness of Hydroxychloroquine and Omalizumab in Chronic Spontaneous Urticaria: A Real-World Study. J. Allergy Clin. Immunol. Pract..

[236] Allison A.C. (2005). Mechanisms of action of mycophenolate mofetil. Lupus.

[237] Shahar E., Bergman R., Guttman-Yassky E., Pollack S. (2006). Treatment of severe chronic idiopathic urticaria with oral mycophenolate mofetil in patients not responding to antihistamines and/or corticosteroids. Int. J. Dermatol..

[238] Zimmerman A.B., Berger E.M., Elmariah S.B., Soter N.A. (2012). The use of mycophenolate mofetil for the treatment of autoimmune and chronic idiopathic urticaria: Experience in 19 patients. J. Am. Acad. Dermatol..

[239] Behrend M. (2001). Adverse gastrointestinal effects of mycophenolate mofetil: Aetiology, incidence and management. Drug Saf..

[240] Steinweg S.A., Gaspari A.A. (2015). Rituximab for the Treatment of Recalcitrant Chronic Autoimmune Urticaria. J. Drugs Dermatol..

[241] Chakravarty S.D., Yee A.F., Paget S.A. (2011). Rituximab successfully treats refractory chronic autoimmune urticaria caused by IgE receptor autoantibodies. J. Allergy Clin. Immunol..

[242] Arkwright P.D. (2009). Anti-CD20 or anti-IgE therapy for severe chronic autoimmune urticaria. J. Allergy Clin. Immunol..

[243] Combalia A., Losno R.A., Prieto-González S., Mascaró J.M. (2018). Rituximab in Refractory Chronic Spontaneous Urticaria: An Encouraging Therapeutic Approach. Ski. Skin. Pharmacol. Physiol..

[244] Mallipeddi R., Grattan C.E.H. (2007). Lack of response of severe steroid-dependent chronic urticaria to rituximab. Clin. Exp. Dermatol..

[245] Rovesti M., Pierobon E., Vaschieri C., Genovese G., Marzano A.V., Lotti T., Satolli F., Feliciani C. (2020). Case of a severe vulgaris and foliaceus pemphigus in a young patient treated with rituximab, with subsequent development of chronic urticaria. Dermatol. Ther..

[246] Asero R., Tedeschi A., Cugno M. (2010). Heparin and tranexamic Acid therapy may be effective in treatment-resistant chronic urticaria with elevated d-dimer: A pilot study. Int. Arch. Allergy Immunol..

[247] Dabas G., Thakur V., Bishnoi A., Parsad D., Kumar A., Kumaran M.S. (2021). Causal Relationship between D-Dimers and Disease Status in Chronic Spontaneous Urticaria and Adjuvant Effect of Oral Tranexamic Acid. Indian Dermatol. Online J..

[248] Dunn C.J., Goa K.L. (1999). Tranexamic acid: A review of its use in surgery and other indications. Drugs.

